# Multi‐Scale Mapping of Gene Expression from Whole‐slide Images for Identifying Phenotype‐Associated Subpopulations

**DOI:** 10.1002/advs.202521151

**Published:** 2026-02-25

**Authors:** Hailong Zheng, Jiajing Xie, Luqi Wang, Hailong Xie, Tong Zhi, Yujia Guo, Bofeng Zhu, Dong Wang, Yu Chen

**Affiliations:** ^1^ Department of Gastroenterology, Digestive Medicine Center, The Seventh Affiliated Hospital Southern Medical University Foshan China; ^2^ Department of General Practice, General Practice Center, The Seventh Affiliated Hospital Southern Medical University Foshan China; ^3^ College of Computer and Information Sciences Fujian Agriculture and Forestry University Fuzhou China; ^4^ Department of Bioinformatics, School of Basic Medical Sciences Southern Medical University Guangzhou China; ^5^ Guangzhou Key Laboratory of Forensic Multi‐Omics for Precision Identification, School of Forensic Medicine Southern Medical University Guangzhou China; ^6^ Key Laboratory of Forensic Medicine in Shanxi Province, School of Forensic Medicine Shanxi Medical University Jinzhong China

**Keywords:** deep learning, gene expression prediction, multi‐scale gene expression, phenotype‐associated subpopulations, whole‐slide images

## Abstract

Discovery of phenotype‐associated subpopulations is critical for targeted therapies and prognostic biomarker discovery, which requires multi‐scale gene expression. Deep learning advancements have enabled cost‐effective genetic alteration inference from whole‐slide images (WSIs), but most methods operate at a single scale. This study presents BiSCALE, a deep‐learning framework that predicts gene expression from WSIs at both tissue (bulk) and near‐cellular (spot) levels and links these predictions to clinical phenotypes. The framework integrates a WSI foundation encoder with a Vision–Mamba fusion module and a two‐stage training strategy to bridge scale and distribution differences between bulk and spot data. Trained on 2109 bulk tumor samples and 141 000 spatial transcriptomics spots across three cancer types, BiSCALE outperforms established bulk and spatial baselines, generalizes well to independent cohorts, and demonstrates strong concordance between predicted bulk and spot expression profiles. It recovers biologically relevant pathway activity and supports downstream applications, including patient‐level risk stratification from bulk WSIs and spot‐level cell‐identity annotation. BiSCALE also identifies phenotype‐associated subpopulations, including niches linked to recurrence and hypoxia. These results establish BiSCALE as a cost‐effective approach for multi‐scale gene analysis and phenotype‐associated feature discovery from routine pathology. All code used in this study are available at: https://github.com/Hailong‐Zheng/BiSCALE.

## Graphical abstract

BiSCALE: A pathology‐driven deep learning framework for multi‐scale gene expression prediction from whole‐slide images. It accurately infers bulk and near‐cellular spot‐level expression, links predictions to clinical phenotypes, identifies disease‐associated niches, and enables applications in risk stratification and cell‐identity annotation, providing a cost‐effective tool for molecular phenotyping.

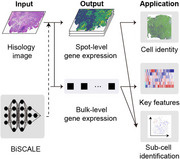

## 1 Introduction

Identifying the cellular subpopulations that drive key clinical phenotypes, such as survival, and therapeutic response, is essential for developing targeted cell therapies and discovering prognostic biomarkers. Here, cellular subpopulations refer to relatively homogeneous subsets of cells within a given canonical cell type, further stratified by transcriptomic features that reflect distinct functional states. For example, prior studies have delineated highly malignant, cancer stem–like tumor‐cell subpopulations that underlie recurrence and poor prognosis [1, 2], uncovered dendritic‐cell subsets that potentiate anti‐tumor immunotherapy responses [3], and identified fibroblast subtypes associated with chemoresistance and disease progression [4].

The prevailing strategy for subpopulation discovery relies on single‐cell or spatial transcriptomic profiling, followed by unsupervised clustering to define cell clusters and marker genes and then infer cell identities [5, 6]. However, the widespread application of these technologies in large clinical cohorts remains limited. Most published datasets include fewer than 20 patients, constraining statistical power and hindering robust identification of phenotype‐associated subpopulations [7, 8, 9]. An alternative approach matches single‐cell expression profiles with bulk tissue transcriptomes containing clinical phenotypes to infer relevant subpopulations [10, 11], but this approach still requires costly, multi‐scale gene‐expression assays and complex data generation pipelines.

In contrast, whole‐slide images (WSIs) are common in clinical workflows, readily obtainable at scale, and richly annotated with outcomes such as stage, survival, and treatment response. Beyond serving as a record of tissue morphology, WSIs encode subtle yet systematic architectural and cytologic patterns that reflect underlying molecular programs, including transcriptional states, genomic alterations, and pathway activities [12, 13]. These observations collectively suggest that WSIs can act as a cost‐effective alternative for molecular profiling, provided that appropriate computational models are available.

Building on this premise, recent work in machine learning has begun to explicitly estimate wide panels, or even genome‐wide gene‐expression profiles, directly from WSIs [12, 14, 15, 16]. At the bulk level, transformer‐based models (e.g., tRNAsformer) leverage self‐attention to capture contextual inter‐tile interactions and predict bulk gene‐expression profiles from slides [17]. SEQUOIA further integrates paired WSIs with bulk expression using a linear transformer to recover cancer‐wide transcriptomic profiles [18]. While powerful, bulk‐level models do not explicitly resolve intra‐tumoral heterogeneity. With the rise of spatial transcriptomics, methods such as ST‐Net [19], mclSTExp [20], and iStar [21] integrate WSIs with paired spatial data to predict gene expression at near‐cellular (spot) resolution, improving spatially precise phenotype mapping and prognosis. However, the predictive performance at the spot level remains significantly lower than that at the bulk level, making clustering analyses based solely on spot‐level predictions less suitable for identifying phenotype‐associated cell subpopulations. To address this gap, a unified framework that predicts both bulk‐ and spot‐level gene expression from WSIs is needed. Such a framework would combine the inter‐patient heterogeneity captured by bulk data, with the intra‐tumoral heterogeneity revealed by spatial data, to identify phenotype‐associated cell subpopulations from a molecular perspective.

Here, we present **Bi**‐scale **S**lide‐based **C**ontext‐**A**ware **L**earning of **E**xpression (BiSCALE), a deep‐learning framework that predicts gene expression at both the bulk and spot levels from WSIs. To improve generalization, we initialize a WSI foundation model to extract robust histopathological features. We then add a Vision–Mamba [22]–based fusion module that captures long‐range context and accommodates the large patch‐count disparity between clinical WSIs (bulk level) and the WSI patches used in spatial transcriptomics (spot level), enabling accurate multi‐scale gene‐expression inference. BiSCALE is developed on 2109 paired tumor WSIs with bulk expression and 141 000 spatial spots with paired image patches and expression across three cancer types, and it is validated on independent cohorts. Using the resulting multi‐scale predictions, we demonstrate downstream applications at both levels, including identifying phenotype‐associated genes at the bulk level and annotating cell identities at the spot level, which together support the discovery of phenotype‐associated cellular subpopulations. Overall, BiSCALE provides a cost‐effective and scalable approach to infer and analyze gene‐expression programs from routine pathology, linking patient‐level and spatially resolved signals to accelerate biomarker discovery and the development of targeted cellular therapies.

## 2 Results

### 2.1 Overview of BiSCALE

**FIGURE 1:**
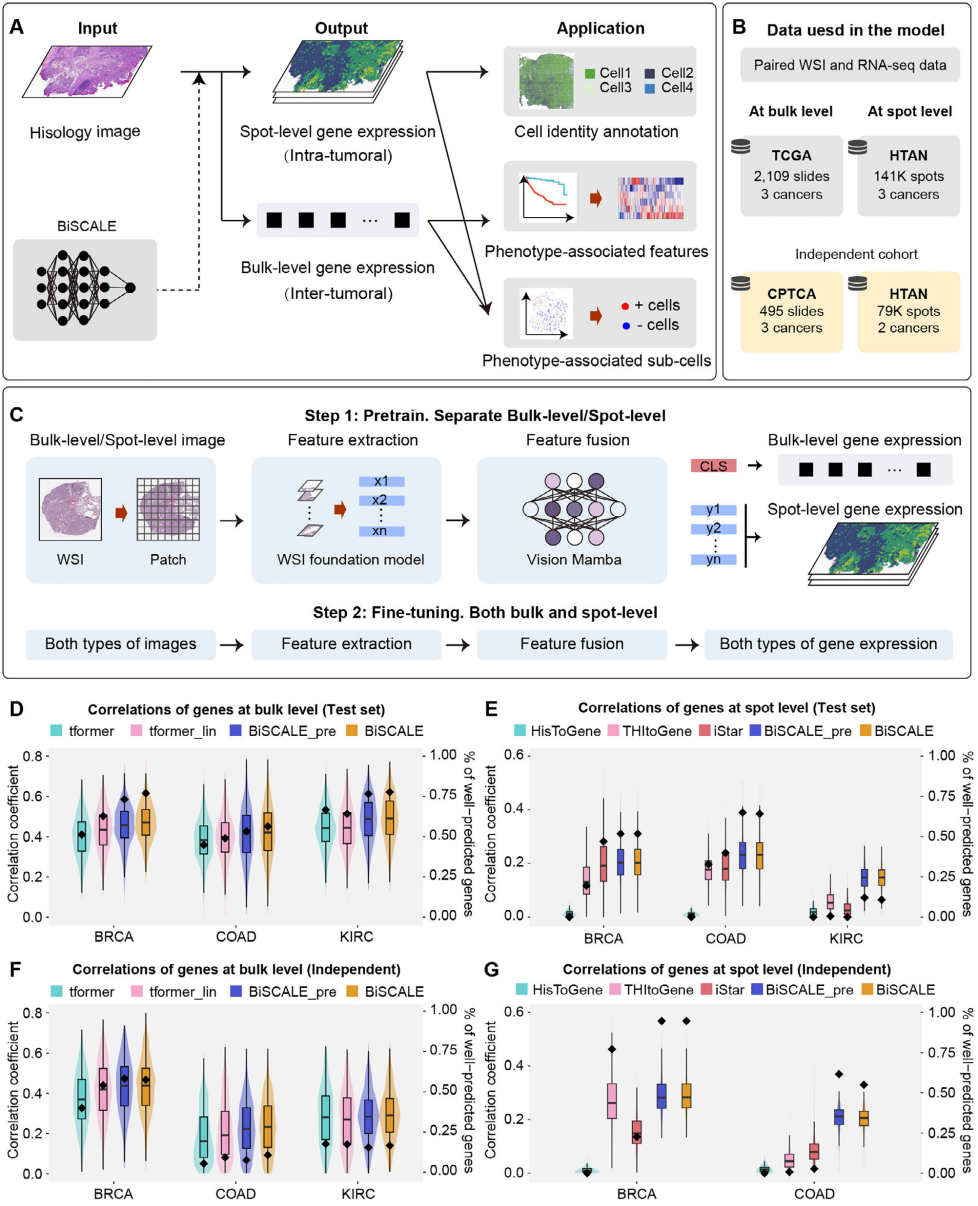
Overview of the BiSCALE workflow. (A) Schematic overview of the BiSCALE framework. First, BiSCALE predicts gene expression from WSIs at both bulk and spot levels. Then, multi‐scale predictions are applied to downstream tasks at each scale, supporting the identification of phenotype‐associated cellular subpopulations. (B) Dataset overview showing matched WSIs and RNA‐seq data at both bulk and spot levels. (C) BiSCALE is trained in two stages: first, pretraining separately on bulk and spot data, followed by joint fine‐tuning on both modalities to reduce scale‐dependent distribution shifts. (D‐G) Violin plots illustrate the distribution of Pearson correlation coefficients (left y‐axis) between predicted and ground‐truth gene expression values in the (D‐E) test set and (F‐G) the independent validation set. Black squares indicate the percentage (right y‐axis) of genes with significantly well‐predicted expression levels. BiSCALE is compared with bulk‐level transformer‐based model (tformer) and linearized transformer‐based model (tformer_lin), as well as spot‐level methods, including HisToGene, THItoGene and iStar, along with BiSCALE's pretraining variant (BiSCALE_pre).

We introduce a two‐step framework to identify phenotype‐associated cell subpopulations from a gene‐level perspective (Figure 1A). First, we developed BiSCALE, a deep‐learning model that infers gene expression from WSI at both the bulk and spot levels. Second, we leveraged similarity in these multi‐scale predictions to identify phenotype‐associated cell subpopulations.

In this study, we trained BiSCALE separately for three cancer types (breast invasive carcinoma, BRCA; colon adenocarcinoma, COAD; kidney renal clear cell carcinoma, KIRC), for which public datasets provide paired WSIs and RNA‐seq at both bulk and spot resolutions (Figure 1B). Bulk‐level data comprised matched WSIs with corresponding bulk RNA‐seq (Table S1), whereas spot‐level data comprised near‐cellular‐resolution spatial transcriptomics with matched histology patches (Table S2). Bulk‐ and spot‐level datasets aggregated from TCGA and HTAN were used for model development and evaluation.

Preprocessing and architectural choices were introduced to enable cross‐scale fusion (Figure 1C; Methods). Bulk RNA‐seq was normalized to transcripts per million (TPM), while spot‐level gene expression was normalized to counts per million (CPM). WSIs were tiled at the same magnification and embedded with pathology foundation model CONCH [23] to produce high‐dimensional representations, yielding a unified input format across scales. We then modified Vision Mamba backbone as a single shared architecture for integrating bulk and spot data. The training of BiSCALE was conducted in two stages: an initial pretraining phase on bulk and spot data separately, followed by a joint fine‐tuning phase on both modalities to reduce scale‐dependent distribution shifts.

Model development and testing were performed within each cancer type using five‐fold cross‐validation. In each fold, slides from 80% of patients were used for training (with 10% of that portion held out for model selection), and the remaining 20% were reserved for testing. Because not all genes are predictable from histology, we restricted training within each cancer type to the top 1000 genes whose expression was most strongly associated with image‐derived features in the training set (Methods). For evaluation, we concatenated per‐gene predictions across all patients/spots, and assessed performance against ground‐truth expression using Pearson correlation and mean squared error (MSE).

As shown in Figure 1D, at the bulk level, the median gene expression correlation was 0.470 for BRCA, 0.421 for COAD, and 0.491 for KIRC. At the spot level, the median correlations were 0.202, 0.231, and 0.147 for BRCA, COAD, and KIRC, respectively (Table S3). Among the 1000 target genes, an average of 70.4% were well‐predicted at the bulk level (*r* > 0.4, *p* < 0.05; Figure 1D and Table S4), whereas 42.3% were well‐predicted at the spot level (*r* > 0.2, *p* < 0.05). To assess the impact of gene coverage, we further trained and evaluated BiSCALE on the top 5000, the top 10 000, and all expressed genes. Across both bulk and spot levels for all three cancer types, the top 1000‐gene subset yielded the highest median correlations (Figure S1A,B). We further repeated the bulk gene prediction experiment with four published pathology encoders chosen based on PathBench [24] (UNI2, mSTAR, GPFM) plus CONCH for direct comparability. The best‐performing encoder differed by cancer type, with GPFM performing best in BRCA (*r* = 0.521), CONCH in COAD (*r* = 0.415), and mSTAR in KIRC (*r* = 0.546) (Figure S1C,D), indicating that no single encoder dominates across cohorts. We therefore used CONCH as a single default encoder for reproducibility and efficiency, given its smallest parameter footprint and lowest computational cost among the evaluated encoders.

We benchmarked BiSCALE against state‐of‐the‐art WSI‐based predictors of gene expression at both the bulk and spot levels across three cancer types. At the bulk level, BiSCALE outperformed transformer‐based tRNAsformer [17] and linear‐transformer‐based SEQUOIA [18], increasing the correlation between predicted and ground‐truth gene expression by 112% and 108%, respectively (Figure 1D and Table S3). These gains were statistically significant within each cancer type (Wilcoxon signed‐rank test, *p* < 0.05; Table S5). Additionally, BiSCALE achieved an average 102% improvement in correlation compared to its pretraining variant without multi‐scale data. This improvement consistently demonstrated higher median correlation of BiSCALE across all cancer types, with statistical significance reached in BRCA (Wilcoxon signed‐rank test, *p* < 0.05; Table S5). At the spot level, BiSCALE was benchmarked against two widely used categories of methods, whole‐image approaches and spot‐conditioned approaches [25, 26]. We selected the top three published, best‐performing baselines from each category for comparison [25]. First, within the whole‐image category, where BiSCALE also belongs, BiSCALE significantly outperformed iStar by an average of 117%, THItoGene by 144%, and HisToGene by >200% in correlation (Figure 1E and Table S3), with substantial improvements across cancer types (Wilcoxon signed‐rank test, *p* < 0.05; Table S5). Notably, in KIRC, BiSCALE maintained a correlation of 0.147, while competing methods yielded near‐zero values. Second, within the spot‐conditioned category, BiSCALE achieved the best performance in BRCA and KIRC, and ranked second in COAD, where it was only surpassed by DeepPT (Figure S2 and Table S3). When compared with the BiSCALE pretraining variant without multi‐scale data, spot‐level improvements were modest across all three cancer types. A plausible explanation is the large size of the spatial‐transcriptomics training set (over 141 000 spots), which likely provided sufficient signal for effective pretraining even without multi‐scale inputs. Analyses based on MSE supported the same conclusions (Figure S3).

To more broadly evaluate cross‐cancer generalizability, we extended the bulk‐level benchmark from three cohorts to all 16 TCGA cancer types for which matched bulk expression data were available (Table S6). Under the same protocol, BiSCALE outperformed both tRNAsformer and SEQUOIA in 13/16 cohorts (Figure S4), yielding a higher median correlation (0.460) than tRNAsformer (0.405) and SEQUOIA (0.410). The three cohorts (PAAD, PRAD, SKCM) where BiSCALE did not lead may reflect instability due to limited sample sizes; for example, PAAD (n = 183) and SKCM (n = 282) are among the three smallest TCGA cohorts in our study.

We further assessed external generalization on independent cohorts by evaluating models trained on TCGA and HTAN on independent test sets, including CPTAC and non‐overlapping HTAN cohorts (Tables S1 and S2). At the bulk level, BiSCALE yielded significantly higher correlation coefficients than all other methods across all three cancer types (Wilcoxon signed‐rank test, *p* < 0.05; Figure 1F and Tables S7 and S8). BiSCALE also achieved a higher median correlation than its pretraining variant, although this difference did not reach statistical significance. At the spot level, BiSCALE consistently achieved an average correlation above 0.2 and significantly outperformed all compared baselines from both whole‐image and spot‐conditioned methods (Figure 1G; Figure S2 and Tables S7 and S8). Collectively, these results indicate that BiSCALE exhibits superior generalization relative to all other methods at both the bulk and spot levels.

Moreover, since cell subpopulations identification depends on bulk–spot expression similarity, we quantified the similarity between BiSCALE's spot and bulk predictions against ground truth (Methods) and compared it with other bulk–spot method combinations. For a fair comparison, we constructed baseline combinations by pairing the top‐performing spot‐level predictors identified in our benchmark (iStar, DeepPT, and EGNv1) with bulk predictors (transformer‐based and linear‐transformer‐based models). Across all cancer types, BiSCALE achieved a mean Area Under Curve (AUC) of 0.776, outperforming all baseline combinations (Figure S5A– C), and slightly exceeding the performance of the pretraining variant of BiSCALE (AUC: 0.769). To test whether these findings based on the 1000 target genes generalize to the full transcriptome (mean 18 622 genes per dataset), we repeated the analysis using all expressed genes as the reference and compared the outcomes. BiSCALE also yielded a higher mean AUC of 0.802 and outperformed all comparator pipelines (Figure S5D–F). Taken together, these results show that BiSCALE provides robust gene‐expression prediction at both the bulk and spot levels, supporting downstream identification of phenotype‐associated cell subpopulations.

### 2.2 Pathway‐Level Evaluation of Predicted Gene Expression

**FIGURE 2:**
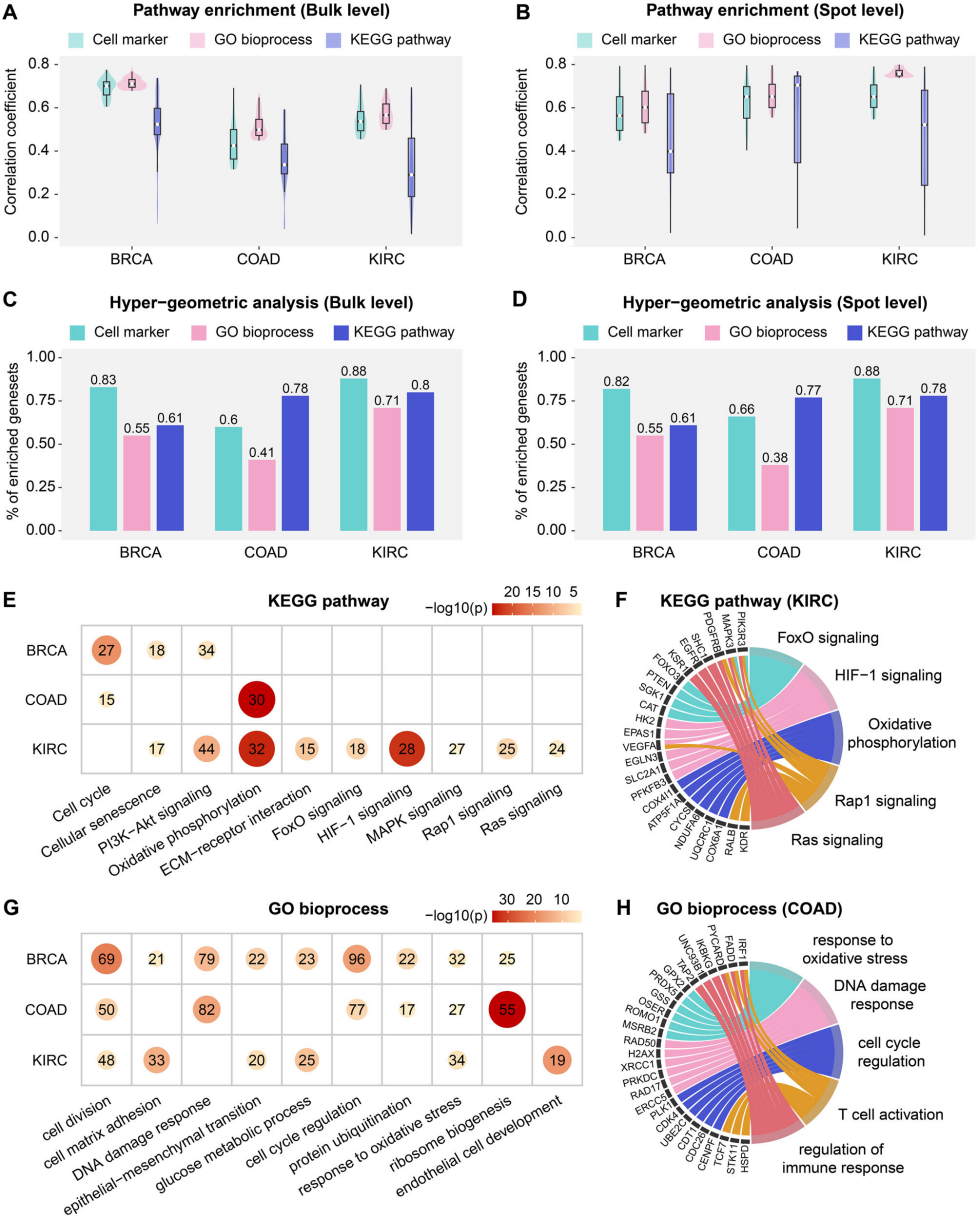
Pathway enrichment analyses of BiSCALE‐predicted gene expression. (A‐B) Violin plots showing the correlation between predicted and true pathway scores, computed using ssGSEA from predicted (A) bulk‐level and (B) spot‐level expression values. The top 100 pathways with the highest correlations from each model are shown. (C‐D) Percentage of enriched pathways recovered by well‐predicted genes from the original 1,000‐gene set. The significance of the enriched pathway was assessed using hypergeometric analysis at (C) bulk‐level and (D) spot‐level. (E, G) Heatmap showing the significance of (E) KEGG pathway enrichment and (G) Gene Ontology enrichment for well‐predicted genes at the bulk level. Circle color and size represent the negative log‐transformed p‐values, and integers indicate gene counts in each pathway. (F, H) Circos plots showing KEGG pathway and GO enrichment with well‐predicted genes in KIRC and COAD, respectively.

To assess whether BiSCALE's predicted expression values capture biological function at both bulk and spot levels, we performed pathway‐level analyses across KEGG pathways, Gene Ontology biological processes, and cell‐type signature sets (Methods). First, we applied single‐sample gene set enrichment analysis (ssGSEA) to compute pathway activity scores from predicted expression values, enabling pathway‐level comparisons with ground truth. Second, within each cancer type, we performed hypergeometric enrichment tests on the well‐predicted genes to assess their coverage across biological pathways. ssGSEA analyses showed that, at the bulk level, the mean correlation coefficient between predicted and true pathway scores was 0.382 for KEGG, 0.395 for GO, and 0.425 for cell‐type signature (Figure 2A). At the spot level, the corresponding mean correlations remained robust at 0.524, 0.306, and 0.394, respectively (Figure 2B). Consistent with these results, hypergeometric tests indicated that, when using pathways enriched by all 1000 genes as a reference, the well‐predicted genes on average covered 73% of KEGG sets, 56% of GO sets, and 77% of cell‐type signature sets (Figure 2C). At the spot level, these averages were 72%, 55%, and 79%, respectively (Figure 2D). Moreover, when we applied the same ssGSEA pipeline to gene‐expression predictions from baseline models, BiSCALE showed the strongest overall pathway‐level concordance at both bulk and spot levels, consistent with the gene‐level comparisons (Tables S9– S11). The only notable exception was COAD at the spot level, where DeepPT was marginally better. Taken together, these results showed that well‐predicted genes cover most functionally important pathways at both bulk and spot levels.

Focusing on the bulk level, we next characterized cancer‐relevant pathways enriched in gene sets from three distinct sources. For KEGG, hypergeometric enrichment revealed that well‐predicted genes were consistently enriched in several shared pathways across cancers, including the Cell cycle (*EGFR*, *ERBB2*, *BCL2*), Cellular senescence (*MYC*, *CCND1*, *TGFB1*), PI3K–Akt signaling (*BMP2*, *PDPN*, *SMAD2*), and Oxidative phosphorylation (*COX4I1*, *ATP5F1A*, *CYCS*) (Figure 2E and Table S10). We also observed cancer‐specific enrichments. For example, in KIRC, the well‐predicted genes were enriched in HIF‐1 signaling (*HK2*, *EPAS1*, *VEGFA*), Ras signaling (*MAPK3*, *KSR1*, *SHC1*), Rap1 signaling (*RALB*, *PIK3R3*, *KDR*), and FoxO signaling (*FOXO3*, *PTEN*, *SGK1*) (Figure 2F). Published studies have revealed the central roles of HIF‐1 signaling in KIRC, which controls angiogenesis, metabolic rewiring, and invasion [27]. Similarly, therapeutic reactivation of FoxO, which is typically inhibited in KIRC through PI3K/AKT/mTOR‐mediated cytoplasmic retention, has been proposed to induce apoptosis and cell‐cycle arrest [28].

Gene ontology enrichment analyses also showed that, across three cancer types, well‐predicted genes were significantly enriched in cell division (*ERCC5*, *CDK4*, *CENPF*) and response to oxidative stress (*GPX2*, *GSS*, *MSRB2*) (Figure 2G and Table S13). In COAD, the well‐predicted genes showed regulation of DNA damage response (*SPHK1*, *H2AX*, *PRKDC*), cell‐cycle regulation (*CDC26*, *CDK4*, *CDT1*), T‐cell activation (*FADD*, *HSPD1*, *IRF1*), and regulation of immune response (*IKBKG*, *PYCARD*, *TAP2*) (Figure 2H and Table S13), consistent with prior studies [29, 30].

In addition, we identified several well‐predicted cell‐type signatures, including dendritic cells (*CD74*, *HLA‐DRA*, *LYZ*), endothelial cells (*CD31*, *CD36*, *VWF*), natural killer cells (*CTSK*, *IGFBP7*, *HLA‐E*), and cancer‐associated fibroblasts (*APOE*, *LGALS3*, *HLA‐DPB1*) (Figure S6A,B and Table S14). These findings indicated that BiSCALE effectively captures key features of the tumor microenvironment.

We further validated these observations in the independent CPTAC cohort by repeating pathway enrichment on well‐predicted genes. The results again highlighted the activity of these genes in cell‐cycle regulation, response to oxidative stress, DNA damage response, and glucose metabolic process (Figure S6C–E). Collectively, enrichment analyses consistently demonstrated that BiSCALE can accurately predict genes central to cancer initiation and progression.

### 2.3 Bulk‐Level Application for Phenotype‐Associated Gene Identification

To motivate analyses leveraging both bulk‐ and spot‐level gene expression, we first demonstrate that BiSCALE's bulk‐level predictions are useful for downstream tasks. Specifically, we evaluated its ability to leverage well‐predicted genes for (i) stratifying clinical phenotypes and (ii) discovering phenotype‐associated genes.

With tumor recurrence as the target phenotype, we trained a regularized Cox proportional‐hazards model on the bulk‐level ground‐truth gene expression for the genes well‐predicted by BiSCALE, and estimated recurrence risk scores for each patient (Methods). For each of the three cancer types, the model was developed in the corresponding TCGA cohort (mean 909 patients) and evaluated in the independent validation dataset (Table S15).

**FIGURE 3:**
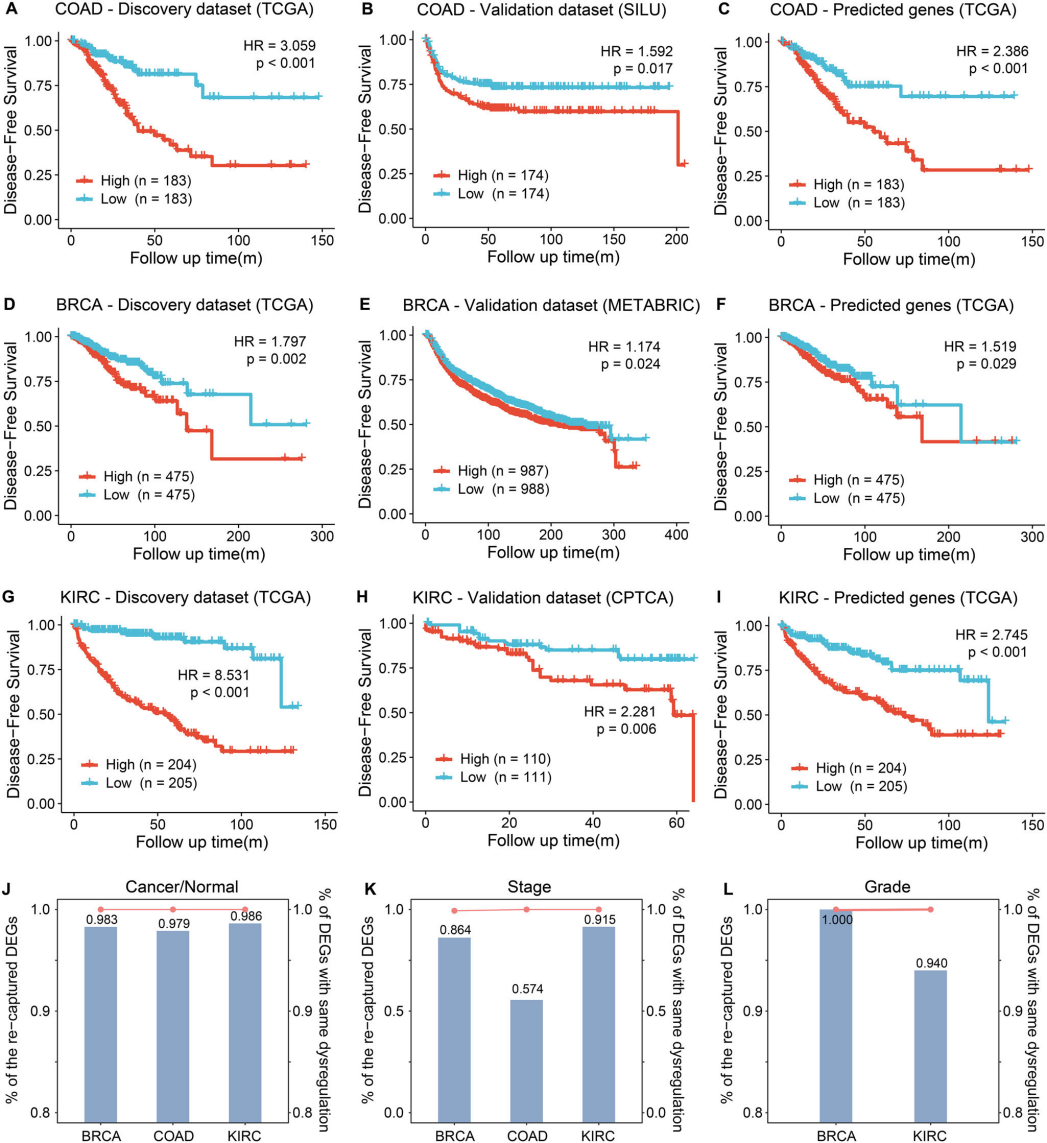
Evaluation of BiSCALE for clinical phenotype stratification and phenotype‐associated gene discovery. (A) Kaplan–Meier curves comparing high‐ and low‐risk groups for recurrence in the TCGA‐COAD cohort. Risk scores were generated from a Cox proportional hazards model trained on bulk RNA‐seq ground‐truth gene expression. (B, C) Validation of the recurrence risk stratification in (B) an independent SILU cohort and (C) the TCGA‐COAD cohort using BiSCALE‐predicted expression. (D–I) Performance of similar Cox model‐based stratification in BRCA (D–F) and KIRC (G–I) cohorts, assessed using ground‐truth expression from TCGA, an independent dataset, and BiSCALE‐predicted expression, respectively. (J–L) Recovery of phenotype‐associated differentially expressed (DE) genes. Bar plots (left y‐axis) indicate the proportion of recovered DE genes, while line plots (right y‐axis) show the concordance rate in the direction of expression change (up/down‐regulated) for these genes.

Using COAD as an example, model training yielded a six‐gene signature associated with recurrence (Table S16). Several signature genes are already known to be associated with COAD recurrence, including *MORC2* [31] and *PYCR2* [32]. To assess risk stratification, we split patients into high‐ and low‐risk groups using the median risk score. In the TCGA discovery set, high‐risk patients exhibited significantly worse outcomes (log‐rank *p* < 0.001, HR = 3.059; Figure 3A), and this separation was observed again in the independent set SILU (log‐rank *p* = 0.017, HR = 1.592; Figure 3B). We further evaluated whether gene expression predicted by BiSCALE is sufficient for risk stratification. For each patient in the TCGA set, we recalculated the Cox risk score with the same coefficients, replacing true expression with BiSCALE predictions. As shown in Figure 3C, the high‐risk group also had significantly shorter recurrence‐free survival (log‐rank *p* < 0.001, HR = 2.388). Similar analyses in BRCA and KIRC also identified prognostic genes via Cox modeling (Table S16), several of which are literature‐supported, including *GLUL* [33] and *CCL19* [34] in BRCA, and *EIF4EBP1* [35] and *SAA1* [36] in KIRC. In both cancer types, risk scores derived in the discovery sets separated patients into two groups with significantly different recurrence risk, both in independent validation datasets and when true expression was replaced with BiSCALE‐predicted expression (Figure 3D–I). Across the three cancer types, BiSCALE consistently produced stronger stratification than baseline methods, reflected by higher hazard ratios and more significant separation (Figure S7). Notably, in BRCA, risk stratification remained significant when using BiSCALE‐predicted expression, whereas the corresponding analyses with baseline‐predicted expression were no longer statistically significant, indicating that BiSCALE better preserves prognostic signals.

BiSCALE was further assessed for identifying phenotype‐associated genes via differential expression (DE) analyses (Methods). Using the DE genes identified from true expression as the reference, we quantified their recovery in BiSCALE‐predicted expression and examined whether the direction of change was preserved. For tumor and normal phenotypes, an average of 98.3% of reference DE genes were also identified using BiSCALE across the three cancer types, with 100% concordance in up/down direction (Figure 3J and Table S17). For the binarized clinical stage and histologic grade, the average recovery rates were 78.4% and 97.0%, respectively, again with 100% concordance in the up/down direction (Figure 3K,L and Table S17).

Collectively, these results indicated that gene expressions predicted by BiSCALE are well‐suited for phenotype‐associated gene discovery. Moreover, BiSCALE‐predicted expression can discriminate clinical phenotypes as well as true expression.

### 2.4 Spot‐Level Application for Cell Identity Annotation

**FIGURE 4:**
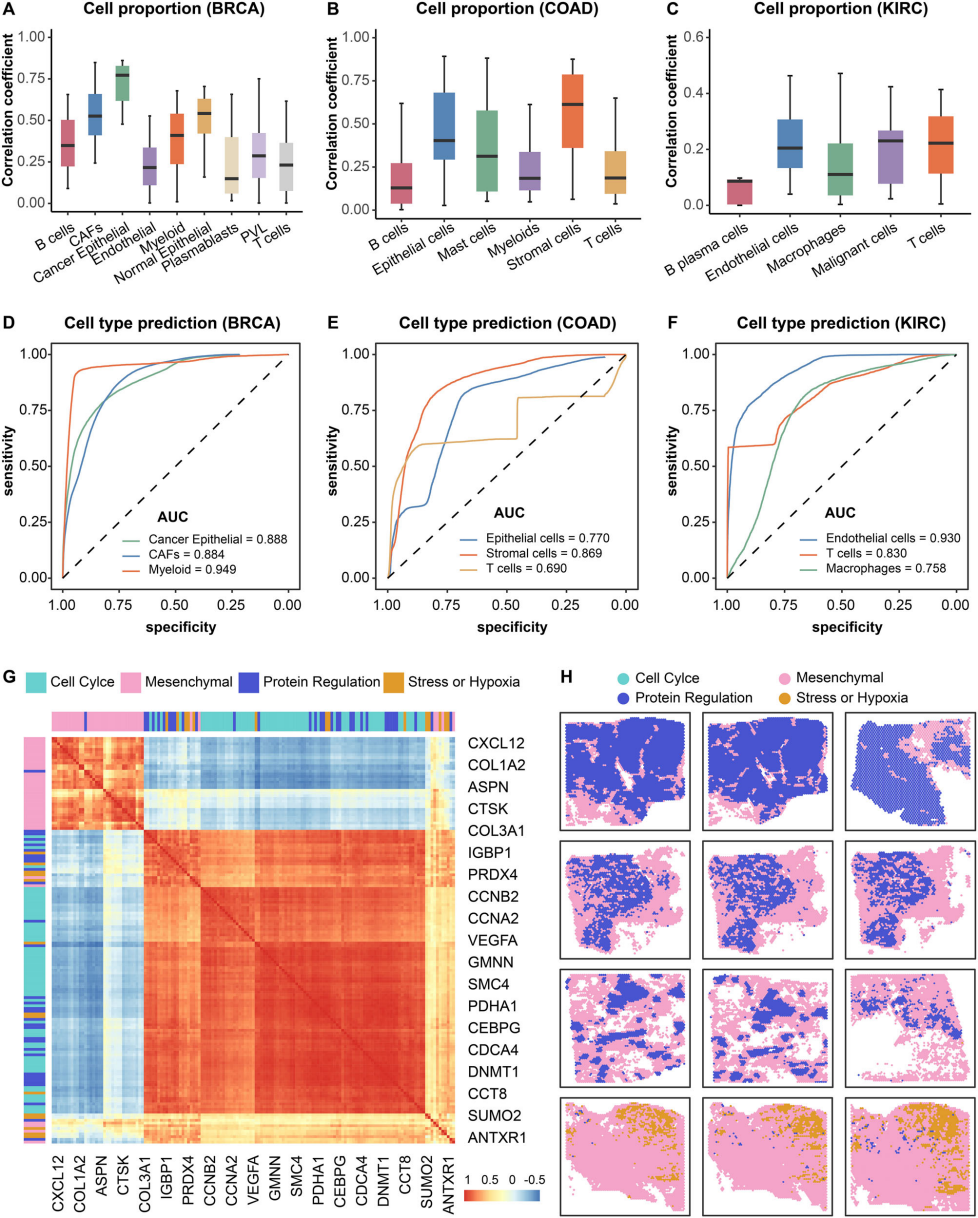
BiSCALE performance in spot‐level cell‐identity annotation and spatial co‐expression analysis. (A–C) Correlation between cell‐type proportions computed from BiSCALE‐predicted spot‐level expression and ground‐truth expression across three cancer types. (D–F) Accuracy of spot‐level cell‐type identification, presented as AUC for the top three cell types. BiSCALE‐based annotations were benchmarked against labels from ground‐truth expression. (G) Heatmap showing the Pearson correlation coefficients for functionally related genes from established meta‐programs of transcriptional intratumor heterogeneity. (H) Spatial maps of gene expression in BRCA spatial transcriptome data. Genes from the same meta‐program formed coherent spatial clusters, with stress/hypoxia genes enriched in the tumor core and mesenchymal genes localized to the invasive margin.

We next evaluated whether spot‐level expression predicted by BiSCALE can be used for downstream tasks, such as cell‐identity annotation. For the spot‐level predicted expression from HTAN (Table S2), we applied the deconvolution method CARD [37] to estimate cell‐type proportions per spot and thereby assign cell identities (Methods). Across all three cancer types, cell‐type proportions inferred from BiSCALE‐predicted expression correlated well with those obtained from ground‐truth expression (Figure 4A–C), with mean correlations of 0.363 (BRCA), 0.237 (COAD), and 0.102 (KIRC). In BRCA, for example, the mean correlation reached 0.657 for the dominant epithelial compartment (Cancer/Normal Epithelial), 0.353 for stromal cells (CAFs, Endothelial), and 0.249 for immune cells (e.g., T cells, B cells) despite their lower abundance (Figure S8A–C). To further assess annotation performance, within each cancer, we labeled each spot by its most abundant cell type and computed AUCs against labels derived from ground‐truth expression. For the top three cell types, BiSCALE achieved mean AUCs of 0.907 (BRCA), 0.776 (COAD), and 0.839 (KIRC) (Figure 4D–F), supporting its suitability for spot‐level cell‐type annotation. We further compared BiSCALE with baseline spot‐level predictors by applying the same CARD‐based pipeline to their predicted expression. Across the three cancer types, BiSCALE yielded the strongest overall agreement with ground‐truth deconvolution, achieving higher AUCs for top‐1dominant cell types than existing whole‐image and spot‐conditioned baselines, with only a minor exception where DeepPT performed slightly better in COAD (Figure S8D).

Prior integrative analyses of scRNA‐seq and spatial transcriptomics show that cells sharing the same transcriptional subtype tend to co‐localize within spatially segmented microenvironments [38, 39]. To test whether BiSCALE captures such biological structure, we examined spatial co‐expression of functionally related genes. We focused on four established meta‐programs of transcriptional intratumor heterogeneity encompassing distinct cellular processes: cell cycle, stress/hypoxia, mesenchymal, and protein regulation [40] (Methods). Spatial correlation analysis indicated that genes within the same meta‐program clustered consistently across all three cancers (Figure 4G and Figure S9). In BRCA, spatial maps further illustrated that genes belonging to the same module formed coherent clusters (Figure 4H); for instance, stress/hypoxia programs concentrated toward tumor cores, whereas mesenchymal programs tended to localize at tumor margins.

### 2.5 Identification of Phenotype‐Associated Cell Subpopulations

**FIGURE 5:**
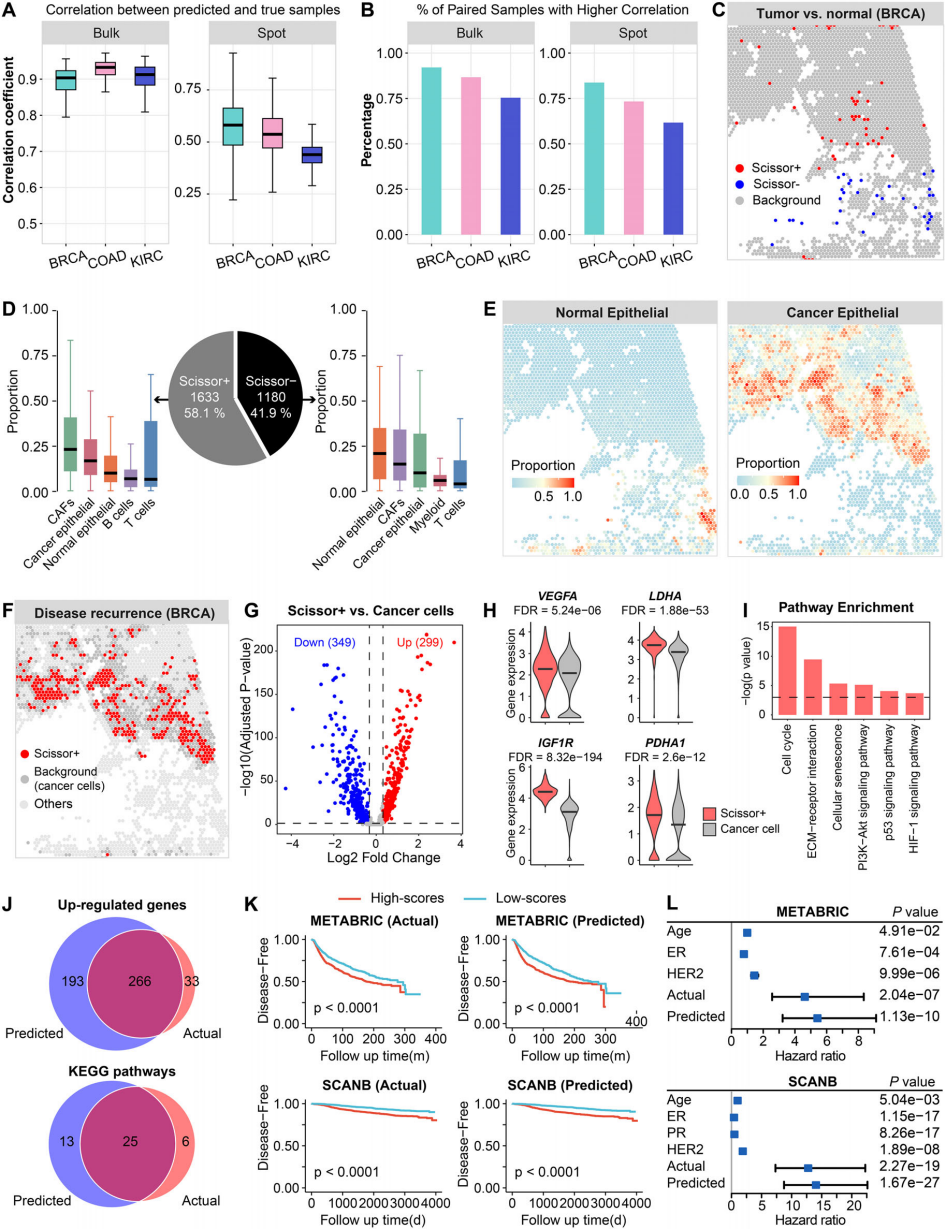
BiSCALE performance in phenotype‐associated subpopulation identification. (A) Sample correlation between predicted and true expression at both bulk and spot levels across all three cancer types. (B) Percentage of cases where the correlation between paired predicted and ground‐truth samples exceeds that of non‐paired samples. (C) Spatial maps of Scissor‐derived, phenotype‐associated subpopulations (Scissor+: tumor‐associated; Scissor‐: normal‐associated) in BRCA using predicted expression. (D) The pie chart of the Scissor‐selected cells with the corresponding bar plots showing the cell type‐specific proportions based on ground‐truth expression. (E) Reference spatial maps of cancer epithelial and normal epithelial cell distribution based on ground‐truth expression. (F) Spatial identification of recurrence‐associated subpopulations using predicted expression. (G) Volcano plot showing DE genes between Scissor+ spots and all other cancer‐cell spots. (H) Violin plot showing the upregulated hypoxia‐related genes in Scissor+ spots. (I) Pathway enrichment analysis on upregulated genes in Scissor+ spots. (J) Venn diagram showing the overlap of DE genes and enriched pathways between predicted and true expression. (K) Patient stratification by prognostic scores (from predicted and true expression) in independent cohorts (METABRIC and SCANB). (L) Forest plot showing univariate Cox regression analysis of the prognostic scores in independent cohorts.

Building on BiSCALE's utility at both scales, we further showed its applicability to identify clinical phenotype–associated cell subpopulations by leveraging correlations between bulk‐ and spot‐level expression. Both bulk and spot expressions were predicted from WSIs, with bulk‐level data sourced from the TCGA dataset and spot‐level data from the HTAN spatial transcriptomics dataset. First, we assessed sample‐level correlations between predicted and true expression at both scales. Across all three cancers, the mean correlation was 0.917 for the bulk level and 0.519 for the spot level (Figure 5A). To examine preservation of tumor heterogeneity in predicted expression, we evaluated whether each predicted sample correlates more strongly with its paired ground‐truth sample than with non‐paired samples. In bulk data, paired correlations exceeded non‐paired correlations in 92.1% (BRCA), 86.7% (COAD), and 75.4% (KIRC) of comparisons; in spot data, the corresponding fractions were 83.7%, 73.3%, and 61.7% (Figure 5B). Based on these results, we conducted two case studies to evaluate BiSCALE's ability to identify phenotype‐associated subpopulations using expression similarity.

First, using tumor/normal labels from 1,204 TCGA‐BRCA bulk samples as the target phenotype, we applied Scissor [11], an approach leveraging bulk–spot gene‐expression similarity, to BiSCALE's predicted gene expression to identify phenotype‐associated subpopulations (Methods). Among the 75 739 spots from 27 spatial transcriptomics samples, Scissor selected 1633 Scissor+ (tumor‐associated) and 1180 Scissor‐ (normal‐associated) spots (Figure 5C). We evaluated the reliability of these identified spots from predicted expression in two complementary ways. First, we performed deconvolution using their ground‐truth expression to estimate the proportions of tumor cells and tumor‐microenvironment cell types. As shown in Figure 5D, cancer epithelial and cancer‐associated fibroblasts were the most abundant populations in Scissor+ spots, whereas normal epithelial cells dominated Scissor‐ spots. Similar composition patterns were reproduced when using the deconvolution results based on predicted expression (Figure S10A), supporting the feasibility of using predicted expression profiles for downstream cell‐type composition analyses. Spatial maps based on true expression showed cancer epithelial enrichment co‐localizing with Scissor+ regions and normal epithelial enrichment co‐localizing with Scissor‐ regions (Figure 5E); the same organization was recovered using predicted expression (Figure S10B). Second, we ran Scissor directly on the ground‐truth spot‐level expression and compared the resulting subsets with those derived from predicted expression. This comparison yielded F1‐scores of 0.94 for Scissor+ and 0.92 for Scissor‐ cells, indicating high consistency between the two labels.

Given the high heterogeneity of cancer cells and the presence of aggressive subsets (e.g., cancer stem‐like cells), we next used disease‐recurrence information from 1009 TCGA‐BRCA bulk samples as the phenotype and applied Scissor to the 3888 cancer‐cell–labeled spots from the same BRCA spatial transcriptomics dataset. The used bulk and spot expressions were also predicted by BiSCALE from the matched WSIs. Scissor identified 350 Scissor+ spots associated with worse outcomes and concentrated in tumor cores, and 44 Scissor‐ spots associated with better outcomes (Figure 5F). To evaluate biological consistency, we compared true expression in Scissor+ spots vs. all other cancer‐cell spots, identifying 299 upregulated and 349 downregulated genes (Figure 5G and Table S18; Methods). Notably, hypoxia‐related genes such as *VEGFA* and *LDHA* were among the upregulated set (Figure 5H), and pathway analysis indicated activation of HIF‐1 and PI3K–Akt signaling in Scissor+ spots (Figure 5I). Beyond subpopulation discovery, we demonstrated the ability of BiSCALE's predicted expression to recover the key genes of these subpopulations. The DE genes and enriched pathways called from predicted expression showed significant overlap with those derived from true expression (hypergeometric test, *p* < 0.05; Figure 5J). To assess clinical relevance, we computed ssGSEA scores for the top‐100 upregulated genes (by fold change) and split patients at the median into high‐ and low‐score groups. As shown in Figure 5K, prognostic scores derived from predicted expression, like those from true expression, separated patients into two groups with significantly different outcomes in both independent cohorts (METABRIC [41] and SCANB [42]). Additionally, univariable Cox analyses showed that, in both independent cohorts, the prognostic score from true expression was a significant risk factor, comparable to the established marker (e.g., *HER2*) and with a higher hazard ratio, which was also replicated when the score was computed from predicted expression (Figure 5L).

Together, these results showed that BiSCALE's WSI‐derived expression not only enables the identification of phenotype‐associated cell subpopulations but also supports downstream molecular feature discovery with spatial coherence and clinical relevance.

## 3 Discussion

In this study, we introduce BiSCALE, a pathology‐driven, multi‐scale framework that predicts gene expression from WSIs at both the bulk and spot levels and uses these predictions to recover phenotype‐associated cell subpopulations. Across three cancers (BRCA, COAD, KIRC), BiSCALE consistently outperformed bulk and spatial baselines, generalized to independent cohorts, and supported downstream analyses that are typically reserved for molecular assays, such as risk stratification at the bulk level and cell‐identity annotation at the spot level. Notably, well‐predicted genes captured core pathways (e.g., cell cycle, PI3K–Akt, oxidative phosphorylation) and tumor–microenvironment signals (e.g., dendritic cells, endothelial cells, CAFs), while bulk–spot concordance enabled identification of tumor/normal‐ and recurrence‐linked subpopulations. Together, these results make BiSCALE a practical bridge between routine histopathology and multi‐scale transcriptomics, expanding opportunities for discovery and translation of valuable biomarkers at the population scale.

Bulk‐level data aggregates slide context and inter‐patient heterogeneity, whereas spot‐level data retains spatial structure and intra‐tumoral diversity. Current existing studies predict gene expression from WSIs at a single scale [12, 14, 15, 16]; in contrast, BiSCALE integrates both scales within one framework. Moreover, its Vision–Mamba fusion and two‐stage training reduce the scale and distribution gap between bulk and spot data, yielding competitive accuracy at both scales and robust cross‐cohort performance. In turn, the high agreement between predicted bulk and spot profiles provides a reliable way for aligning cellular niches with clinical phenotypes. This dual view helps move beyond single‐modality pipelines—bulk‐only models miss spatial ecology, while spot‐only models can be data‐sparse—to a unified analysis that links patient outcomes to localized cellular programs.

Pathway‐level concordance between predicted and true expression underscores that BiSCALE recovers functional signals rather than coincidental correlations. Enrichment of HIF‐1 and mesenchymal programs in recurrence‐linked regions and the coherent spatial clustering of stress/hypoxia modules illustrate that the model preserves biologically meaningful spatial organization. In clinical practice, WSI‐based bulk predictions enabled Cox models to flag high‐risk patients in external cohorts with performance close to that of models using true expression, suggesting a scalable alternative when sequencing is not feasible. At the spot level, CARD‐based deconvolution on predicted expression produced cell‐type proportions and AUCs that agreed with ground truth, enabling routine WSI data to approximate tasks often requiring spatial transcriptomics.

Several limitations of this study are also noted. First, owing to data availability constraints, we evaluated only three cancer types. Additional diseases and tissue types are needed to assess generalizability and domain shift. Second, spot‐level prediction remains more challenging than the bulk‐level counterpart. This can be attributed to the inherent limitations of the data, including sparse counts and a lower per‐spot signal‐to‐noise ratio. Future work could incorporate uncertainty estimation or calibrated denoising to temper downstream inferences. Third, model training focused on the top 1000 image‐predictable genes per cancer, which improves learning stability but may bias analyses toward morphology‐coupled programs; expanding gene coverage with multi‐task objectives is a natural extension. Finally, our validation is retrospective; prospective and multi‐center studies are needed to confirm clinical utility and workflow fit.

In the future direction, BiSCALE can be strengthened by (i) larger foundation models and self‐supervised pretraining on multi‐institutional WSIs, (ii) domain adaptation to harmonize staining and scanner effects, (iii) joint learning with radiology, proteomics, or genomics to enhance pathway fidelity. Further, embedding BiSCALE into pathology pipelines could enable risk scoring and microenvironment readouts at scale, with cost profiles aligned to routine care.

In summary, BiSCALE demonstrates that routine WSIs can yield robust, multi‐scale transcriptomic profiles that generalize across cohorts, recover biologically coherent pathways and niches, and enable phenotype‐driven discovery at both patient and cellular scales. This capability opens a path toward scalable molecular phenotyping from standard‐of‐care histology and toward integrative biomarkers that unite patient‐level outcomes with spatially resolved tumor ecology.

## 4 Methods

### 4.1 Data Sources and Description

To enable model training and independent assessment of generalization, we collected paired WSIs and transcriptomic profiles at two scales, including tissue (bulk) level and near‐cellular (spot) level. We focused on three cancer types for which public data exist at both scales: BRCA, COAD, and KIRC.

#### 4.1.1 Training Datasets

The bulk‐level cohort comprised formalin‐fixed, paraffin‐embedded diagnostic WSIs and matched bulk RNA‐seq from TCGA (https://portal.gdc.cancer.gov) (Table S1); only primary tumor diagnostic slides were included. The spot‐level cohort comprised multimodal 10× Genomics Visium v1.0 datasets curated from the Human Tumor Atlas Network (HTAN) (https://data.humantumoratlas.org), which include spot‐level RNA‐seq and paired histology images (Table S2).

#### 4.1.2 Independent Validation Datasets

To assess external performance, we collected independent validation cohorts at both scales (Tables S1 and S2). At the bulk level, for each cancer type, we sourced paired WSIs and bulk RNA‐seq from the Clinical Proteomic Tumor Analysis Consortium (CPTAC) (https://portal.gdc.cancer.gov). At the spot level, we reserved additional HTAN 10x Genomics Visium v1.0 datasets that were not used for training.

### 4.2 Pre‐Processing of Bulk‐ and Spot‐Level Multimodal Data

#### 4.2.1 RNA‐Seq Normalization

To enable multi‐scale fusion and prediction, gene expression values were normalized consistently across both scales. Bulk RNA‐seq counts were converted to TPM. For 10× Genomics Visium data, spot‐level counts were converted to CPM. In addition, for each spatial transcriptomics sample, we generated a pseudo‐bulk profile by aggregating spot‐level gene counts and converting them to CPM. This pseudo‐bulk CPM profile served as the bulk‐level target for multi‐scale learning, which has been reported to yield results largely concordant with TPM in tasks such as stable‐gene selection and model fitting [43, 44]. All expression matrices (bulk TPM and spot/pseudo‐bulk CPM) were transformed using (v→log2(v+1)), which brings them into a similar dynamic range and facilitates joint training of a single regression model under the MSE loss. In addition, predictions were restricted to protein‐coding genes.

Further, we hypothesized that only a subset of genes is predictable from histology. Accordingly, within each cancer type, we computed Pearson correlations between image‐derived features from the pathology foundation model and gene expression measured at both the spot and bulk levels. For each gene, we ranked the correlation coefficients within each data level and averaged the two ranks; the 1000 genes with the highest mean rank were retained as the target gene set. For the spot‐level analyses, we further restricted to spots with quantified expression for at least 100 genes to ensure sufficient signal.

#### 4.2.2 WSI Preprocessing

WSIs were downloaded in SVS or TIFF format and down‐sampled to 20× magnification (0.5 μm/pixel). To capture morphology consistently across scales, slides were tiled as follows: at the bulk level, each WSI was partitioned into non‐overlapping 256 × 256‐pixel patches (128 μm × 128 μm) and ordered by (x, y) coordinates for sequence modeling; at the spot level, for each Visium spot, a 256 × 256 pixel patch centered on the spot coordinate was cropped from the corresponding WSI. Patches with <15% tissue coverage were discarded to minimize background, and only samples containing more than 1000 tiles were retained. The retained *n* patches were then embedded with the CONCH pathology foundation model [36], yielding an *n* × 512 matrix of patch embeddings used as model inputs.

### 4.3 Construction of a Pathology‐Driven Multi‐Scale Gene‐Expression Predictor

Compared with conventional Transformer‐based models, Mamba‐based models offer linear‐time sequence modeling and are well‐suited to long patch sequences in high‐resolution WSIs. Building on this, we adapt Vision Mamba as a feature‐fusion backbone to process both tissue‐level WSI patch sequences and spot‐level patches from spatial transcriptomics. Because bulk and spot‐level expression distributions differ markedly (e.g., sparsity at the spot level), uninformed joint training can suffer from distribution mismatch. We therefore adopt a two‐stage strategy (Figure 2): pretraining at each scale followed by multi‐scale fine‐tuning.

#### 4.3.1 Pretraining

For a preprocessed WSI, let $X=[x_{1},x_{2},\text{…},x_{n}]$ denote the sequence of n patch embeddings ($x_{i}\in R^{512}$, where *i* = 1, 2, …, n). Vision Mamba was used to fuse patch features through the first‐order causal recurrence equation, which updates the hidden state and generates the output:

(1) $$\begin{array}{c} h_{i}=a_{i}⊙\;h_{i-1}+b_{i}⊙\;x_{i} \end{array}$$

(2) $$\begin{array}{c} y_{i}=g_{i}⊙\left(c_{i}⊙\;h_{i}\right) \end{array}$$

where $x_{i}$, $h_{i}$, and $y_{i}$ are the input, hidden state, and output for the *i*‐th patch; $a_{i}$, $b_{i}$, $c_{i}$ and $g_{i}$ are memory‐decay, input‐injection, read, and gating coefficients, respectively, obtained via learned linear transformations of $x_{i}$:

(3) $$a_{i},b_{i},c_{i},g_{i}=\text{Linear}\left(x_{i}\right)$$

Additionally, a learnable CLS token is appended to the sequence to aggregate global information:

(4) $$\left[y_{1},y_{2},\text{…},y_{n},y_{CLS}\right]=VisionMamba\left(x_{1},x_{2},\text{…},x_{n},CLS\right)$$

Local $\mathrm{outputs}\;\left[y_{1},y_{2},\text{…},y_{n}\right]$ and the global output $y_{CLS}$ (all 512D) are mapped by corresponding MLP heads to gene‐expression predictions:

(5) $${\hat{z}}_{i}=\text{MLP}\left(y_{i}\right),\;i=1,\text{…},n$$

(6) $${\hat{z}}_{\text{global}}=\text{MLP}\left(y_{CLS}\right)$$

Using MSE, the local (spot‐level) and global (bulk‐level) losses are as follows:

(7) $${\text{Loss}}_{\text{local}}=\frac{1}{n}\sum_{i=1}^{n}{\left|{\hat{z}}_{i}-z_{i}\right|}^{2}$$

(8) $${\text{Loss}}_{\text{global}}={\left|{\hat{z}}_{\text{global}}-z_{\text{global}}\right|}^{2}$$

where $z_{i}$ and $z_{global}$ denote the ground‐truth spot‐level and bulk‐level expression, respectively. Pseudo‐bulk RNA‐seq profiles derived from spatial transcriptomics data and conventional bulk RNA‐seq data are used to optimize the global loss ${\mathrm{Loss}}_{global}$, while spot‐level RNA‐seq data are used to optimize the local loss ${\mathrm{Loss}}_{local}$.

During pretraining, we trained two separate models to account for the substantial distributional differences between bulk‐level and spot‐level data. For bulk‐level RNA‐seq, including conventional bulk RNA‐seq and pseudo‐bulk profiles, one model was pretrained with the global loss function ${\mathrm{Loss}}_{bulk}={\mathrm{Loss}}_{global}$. For spatial transcriptomics datasets, which provide both CPM‐normalized spot‐level profiles and pseudo‐bulk profiles in summed CPM, a separate model was pretrained with the combined loss function ${\mathrm{Loss}}_{spot}\;=\;{\mathrm{Loss}}_{local}+{\mathrm{Loss}}_{global}$, which focuses more on the spot‐level data due to their substantial numerical advantage.

#### 4.3.2 Multi‐Scale Fine‐Tuning

During the fine‐tuning stage, we started from the two distinct pretrained models, each focused on either bulk‐level or spot‐level data. Each model was jointly fed tissue‐level samples and spatial transcriptomics data to perform concurrent gene expression prediction, thereby facilitating cross‐scale feature sharing. To enable effective joint training, the spatial transcriptomics samples were up‐sampled to an integer multiple of the tissue‐level sample count. This ensured that each training step could process one spatial sample alongside one tissue‐level sample. The loss functions used for fine‐tuning the two models, ${\mathrm{Loss}}_{bulk}$ and ${\mathrm{Loss}}_{spot}$, are defined as follows:

(9) $${\text{Loss}}_{\text{bulk}}=\lambda \cdot {\text{Loss}}_{\text{local}}+\left(1-\lambda \right)\cdot {\text{Loss}}_{\text{global}}$$

(10) $${\text{Loss}}_{\text{spot}}=\left(1-\lambda \right)\cdot {\text{Loss}}_{\text{local}}+\lambda \cdot {\text{Loss}}_{\text{global}}$$

Here, the hyperparameter λ corresponds to the reciprocal of the up‐sampling factor applied to the spatial transcriptomics samples, serving to balance the contribution of data from different scales. Specifically, λ was set to 0.1 for the BRCA dataset, and to 0.05 for both the COAD and KIRC datasets.

### 4.4 Model Training and Evaluation

We trained cancer–type–specific models using bulk‐level data from TCGA and spot‐level data from HTAN. Model performance was assessed with five‐fold, patient‐wise cross‐validation. In each fold, patients were split into 80% for training and 20% for testing. In addition, we set aside 10% of the training data as an internal validation set to determine the optimal stop point for training. During pretraining, models were pretrained for up to 200 epochs with an MSE‐base loss. For early stopping and determining the point for model saving, we employed a criterion that considers both MSE and Pearson correlation. A checkpoint was saved whenever the MSE‐based loss reached a new minimum. If MSE failed to improve for 20 continuous epochs, training continued only if correlation exceeded its best‐so‐far value. Optimization used Rectified Adam with batch size = 1, learning rate 2 × $10^{-4}$, and weight decay 5 × $10^{-3}$. Each optimization step used one patient‐level sample, either a single bulk sample or one spatial transcriptomic sample comprising multiple spots.

For multi‐scale fine‐tuning, we up‐sampled the pool of spatial transcriptomic samples to an integer multiple of the number of bulk samples so that each optimization step jointly used one spatial transcriptomic sample and one bulk sample. During this phase, early stopping and checkpointing were based only on Pearson correlation, the learning rate was reduced to 2 × $10^{-5}$, and all other hyperparameters were unchanged unless otherwise stated. For final evaluation, Pearson correlation and MSE were computed separately within each of the five test folds and then aggregated across folds to assess performance on the full out‐of‐sample cohort.

### 4.5 Bulk–Spot Expression Similarity Assessment

We evaluated bulk–spot expression similarity using the AUC scores. For *n* spots and *m* bulk samples, we computed all pairwise correlations between BiSCALE‐predicted spot‐ and bulk‐level gene expression, yielding an *n* × *m* correlation matrix. For each spot, we then averaged its correlations across all bulk samples to obtain spot‐level correlation scores. The same procedure was applied to ground‐truth expression, and spots were binarized at the median of their ground‐truth correlations (high vs. low). Using these reference labels, we quantified how well BiSCALE‐based correlations ranked spots by computing the AUC scores.

### 4.6 Gene Set Analysis

We collected gene sets for Gene Ontology (biological process) and cell‐type signatures from MSigDB (https://www.gsea‐msigdb.org/gsea) and KEGG pathway annotations from the KEGG database (https://www.genome.jp/kegg/catalog/org_list.html). For each sample, we computed pathway enrichment scores using ssGSEA implemented in GSEApy (v1.1.9). Further, enrichment scores of all samples were averaged to obtain pathway activity values. In addition, hypergeometric enrichment tests were performed on the well‐predicted genes to assess the significance of their enrichment in each pathway. For each of the three gene‐set sources, we then calculated the percentage of pathways that showed a significant overlap with the well‐predicted genes.

### 4.7 Bulk‐Level Application: Recurrence‐Related Gene Identification

We developed a recurrence risk signature by fitting an L1‐penalized Cox model (LASSO‐Cox) to bulk transcriptomic data from TCGA. The optimal penalty λ was selected by cross‐validation, retaining genes with non‐zero coefficients as candidate prognostic features. A patient‐level risk score is computed as the weighted sum of selected gene expressions:

(11) $$\text{Risk score}=\sum_{i=1}^{n}C_{i}\times {\text{Exp}}_{i}$$

where $C_{i}$ is the Cox coefficient, and $\mathrm{Ex}p_{i}$ is the predicted expression of gene *i*. Patients are stratified into high‐ and low‐risk groups by the median risk score, and differences between the two groups are assessed with the log‐rank test. The finalized model is evaluated on the independent CPTAC cohort to assess generalizability and reproducibility.

### 4.8 Spot‐Level Application: Cell Identity Annotation

We applied CARD [30] on the spot‐level expression predicted by BiSCALE to estimate cell‐type proportions per spot. CARD was run with default settings, using cancer‐type–specific single‐cell RNA‐seq references (Table S17). Only cell types with a proportion >5% were shown. For the three most prevalent cell types in each cancer, we used labels derived from ground‐truth expression as the reference and computed the AUC of the corresponding cell‐type proportions to assess concordance.

### 4.9 Spatial Correlation Analysis of Meta‐Programs

To assess whether genes with similar spatial expression patterns are functionally related, we used four established meta‐programs of transcriptional intratumor heterogeneity that were reported in a published single‐cell RNA‐seq study [33]. For each cancer type, analyses were restricted to the intersection between the 1000 predicted genes and these four gene sets. To quantify spatial co‐expression, we computed pairwise similarities among all genes within the four sets as follows: for a given gene pair, we flattened the spot‐level predictions of each slide into two 1D arrays and calculated the Pearson correlation coefficient. This procedure was repeated across all slides, and the resulting correlation matrices were averaged to obtain a cross‐sample spatial correlation matrix. We then applied hierarchical clustering to this matrix to identify groups of genes with similar spatial patterns and annotated rows and columns with color bars indicating each gene's meta‐program membership.

### 4.10 Phenotype‐Associated Subpopulation Identifications

Leveraging BiSCALE‐predicted bulk‐ and spot‐level expression profiles, we applied Scissor [9] to identify subpopulations whose transcriptomes align with specific clinical phenotypes. We analyzed two phenotype types. First, for the binary tumor/normal phenotype (tumor coded as 1, adjacent normal as 0), Scissor was run on each spatial‐transcriptomics sample, and the regularization parameter was tuned such that approximately 3% of spots were selected as phenotype‐associated, following the threshold used in the original Scissor‐based analysis of these cohorts [11]. The identified Scissor+ spots were interpreted as malignant‐associated, whereas Scissor‐ spots were normal‐associated. Second, for the tumor recurrence phenotype, we focused on the spots labeled with cancer cells by CARD and selected the 10% of these cancer spots as phenotype‐associated, again matching the configuration of the original analysis. In this setting, Scissor+ cells were linked to poorer outcomes, while Scissor‐ cells were linked to favorable outcomes.

### 4.11 Differential Expression Gene Analysis

For bulk‐level gene expression, we performed DE analysis using the Wilcoxon signed‐rank test on binary phenotypes. Notably, the clinical stage was binarized by grouping stages I–II as low stage and stages III–IV as high stage; histologic grade was binarized analogously.

For spot‐level gene expression, DE genes between Scissor+ and Scissor‐ spots were identified with Seurat [45] (FindMarkers, default Wilcoxon rank‐sum test). DE genes were defined by two criteria: FDR < 0.05 and $\left|fold\;change\right|$ > 1.25.

## Author Contributions

H. Zheng, J. Xie, L. Wang, and H. Xie contributed equally to this work. H. Zheng and J. Xie designed and implemented the BiSCALE algorithm. L. Wang, H. Xie, T. Zhi, and Y. Guo collected data and validated the methods. Y. Chen, D. Wang, B. Zhu supervised the project and secured the funding. H. Zheng and J. Xie wrote the original manuscript. All authors read and approved the final manuscript.

## Conflicts of Interest

The authors declare no conflicts of interest.

## Supporting information


**Supporting File**: advs74561‐sup‐0001‐SuppMat.docx

## Data Availability

WSIs, gene expression, and clinical data of TCGA cohorts were retrieved from the publicly available Genomic Data Commons (GDC) portal (https://portal.gdc.cancer.gov). Gene expression data of the CPTAC cohort were downloaded from GDC portal (https://portal.gdc.cancer.gov), and WSIs were obtained from the Cancer Image Archive (https://www.cancerimagingarchive.net/collections). Spatial transcriptomics and matched histology images were obtained from Human Tumor Atlas Network (HTAN) (https://data.humantumoratlas.org/). The RNA‐seq data and clinical annotations of the SCANB, METABRIC, SILU, and CPTAC cohorts were obtained from the cbioportal (https://www.cbioportal.org/datasets). All code used in this study are available at: https://github.com/Hailong‐Zheng/BiSCALE.
