# Supplementary material for: Multi‐Scale Mapping of Gene Expression from Whole‐slide Images for Identifying Phenotype‐Associated Subpopulations
Source: Adv Sci (Weinh). 2026 Feb 25;13(26):e21151. doi: 10.1002/advs.202521151 (PMC13159098; doi:10.1002/advs.202521151)
Supplement: Supplementary file 1 — Supporting File: advs74561‐sup‐0001‐SuppMat.docx [file ADVS-13-e21151-s001.docx]

**Supplementary Information**

Multi-scale Mapping of Gene Expression from Whole-slide Images for Identifying Phenotype-Associated Subpopulations

**This file includes:**

**Supplementary Figures S1 to S10**

**Supplementary Tables S1 to S19**


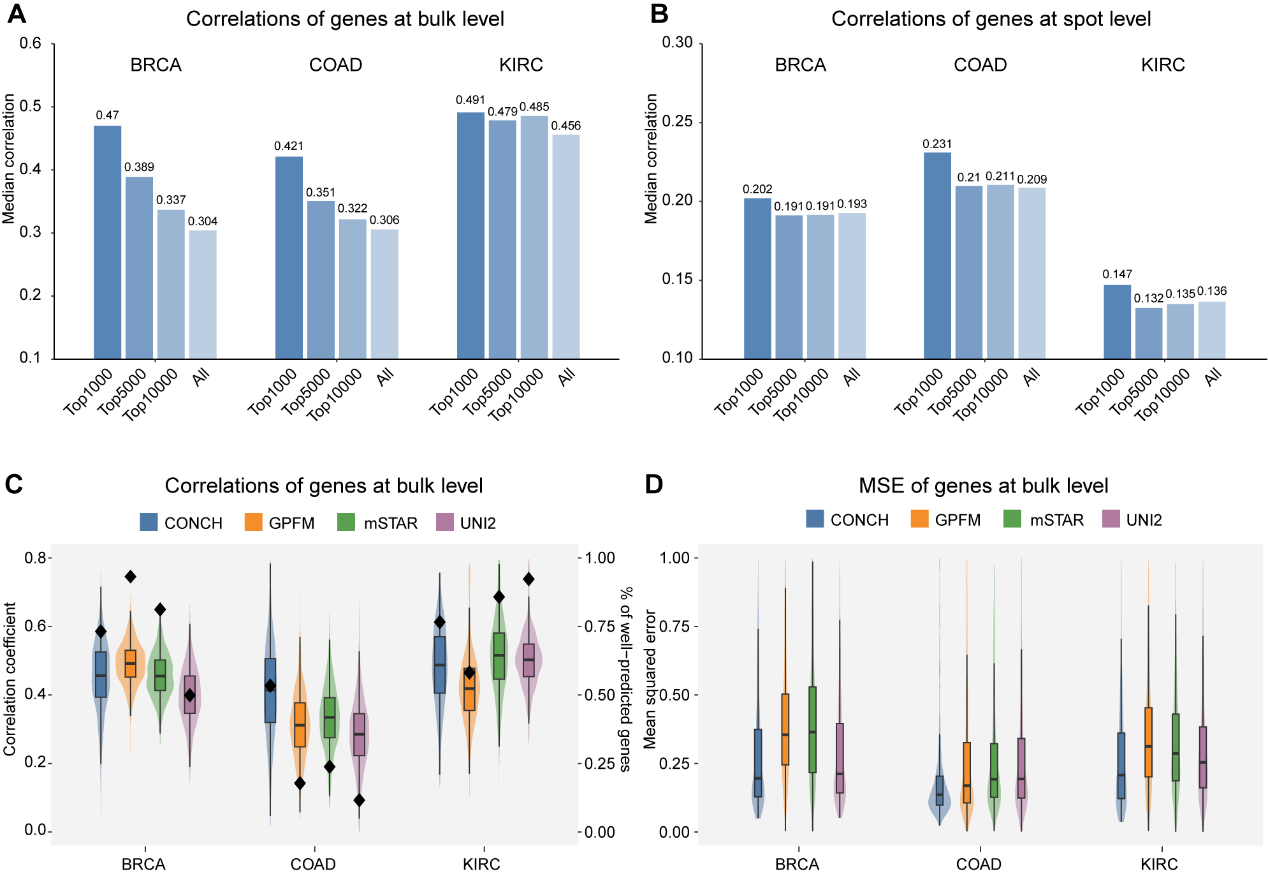


**Figure S1. Sensitivity analyses of BiSCALE gene expression prediction.** (A) Median correlations between BiSCALE-predicted and ground-truth bulk-level gene expression when the model is trained on the top 1,000 genes, the top 5,000 genes, the top 10,000 genes, and all expressed genes. (B) Median correlations between BiSCALE-predicted and ground-truth spot-level gene expression under the same four gene-coverage settings as in (A). (C) Box plots comparing bulk-level prediction performance across pathology foundation encoders (CONCH, GPFM, UNI2, mSTAR) using correlation as the metric. (D) The same comparison as in (C) reported using mean squared error (MSE).


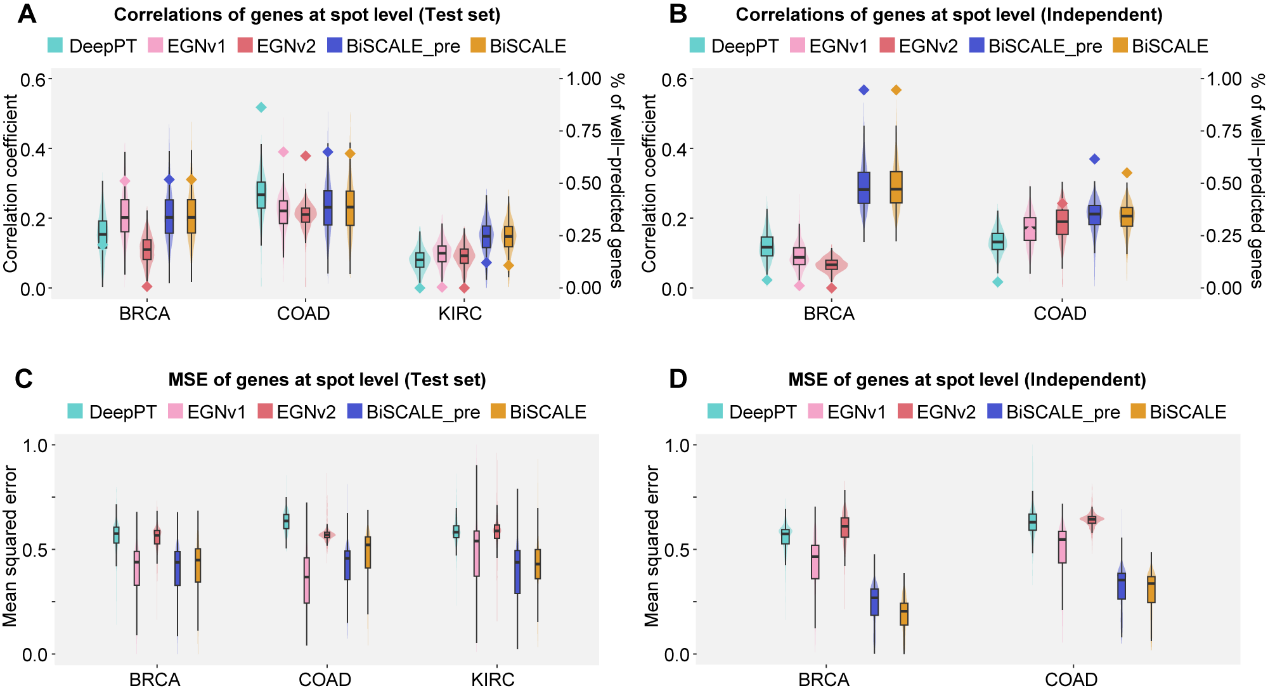


**Figure S2.** **Comparison with spot-conditioned approaches for spot-level gene expression prediction.** (A-B) Violin plots illustrate the distribution of Pearson correlation coefficients (left y-axis) between predicted and ground-truth gene expression values in the (A) test set and (B) the independent validation set. Black squares indicate the percentage (right y-axis) of genes with significantly well-predicted expression levels. (C-D) The corresponding results measured by mean squared error (MSE) in the (C) held-out test set and (D) independent validation set.


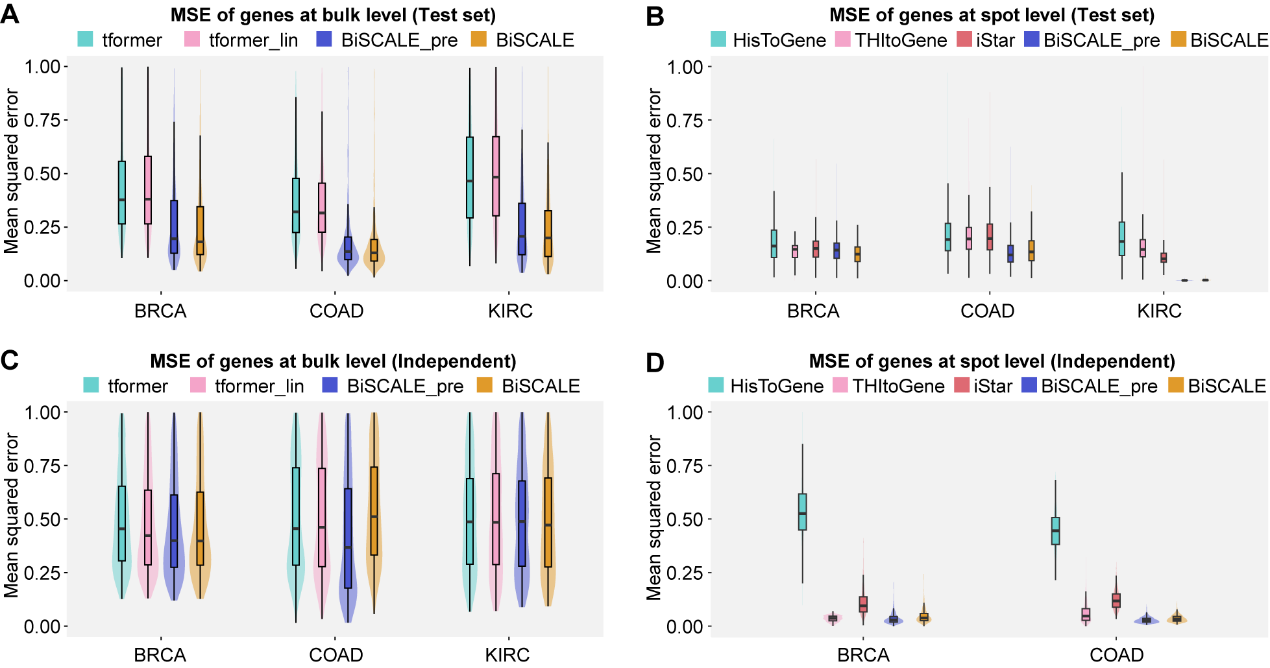


**Figure S3. Performance of BiSCALE in MSE.** Violin plots illustrate the distribution of MSE between predicted and ground-truth gene expression values in the (A-B) test set and (C-D) the independent validation set.


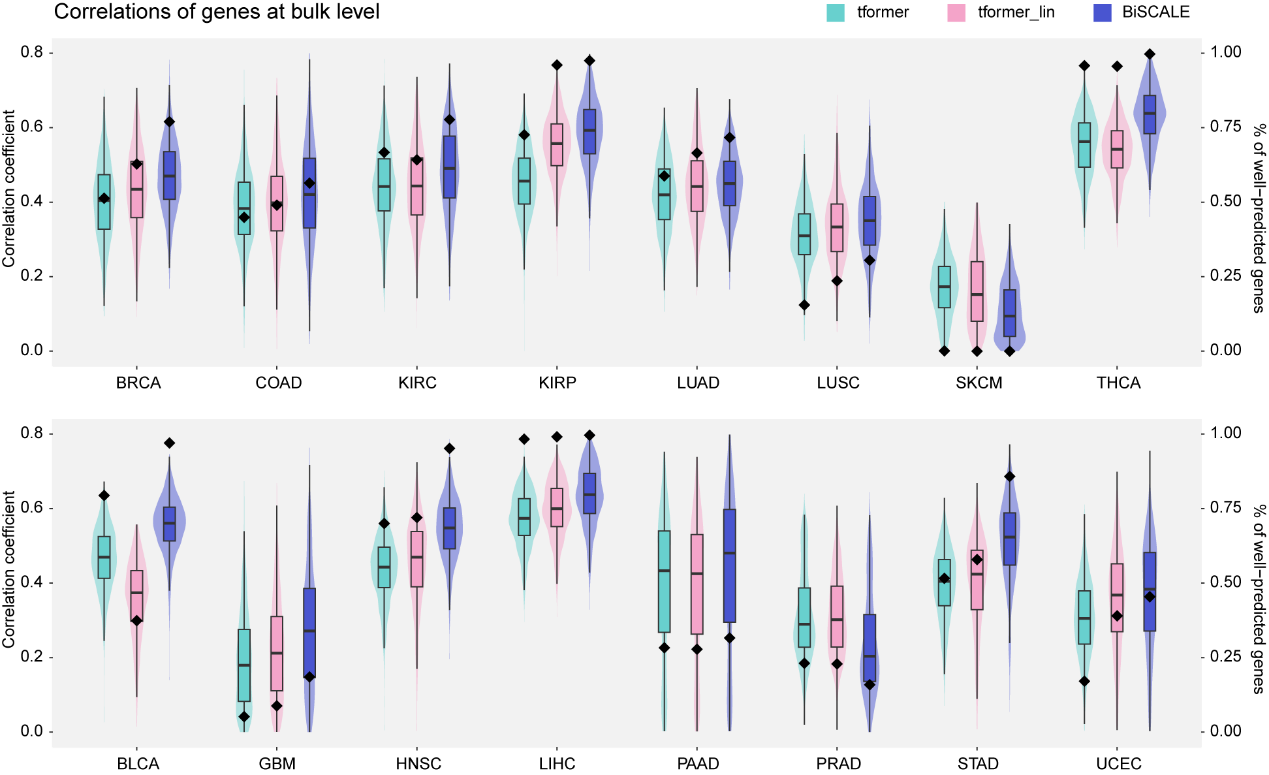


**Figure S4. Cross-cancer generalizability on TCGA bulk gene expression prediction.** Violin plots illustrate the distribution of Pearson correlation coefficients (left y-axis) between predicted and ground-truth gene expression values. Black squares indicate the percentage (right y-axis) of genes with significantly well-predicted expression levels. BiSCALE is compared with bulk-level transformer-based model (tformer) and linearized transformer-based model (tformer_lin). Cancer types are stratified by cohort size, with cohorts of >400 patients shown in the upper panel and cohorts of ≤400 patients shown in the lower panel.


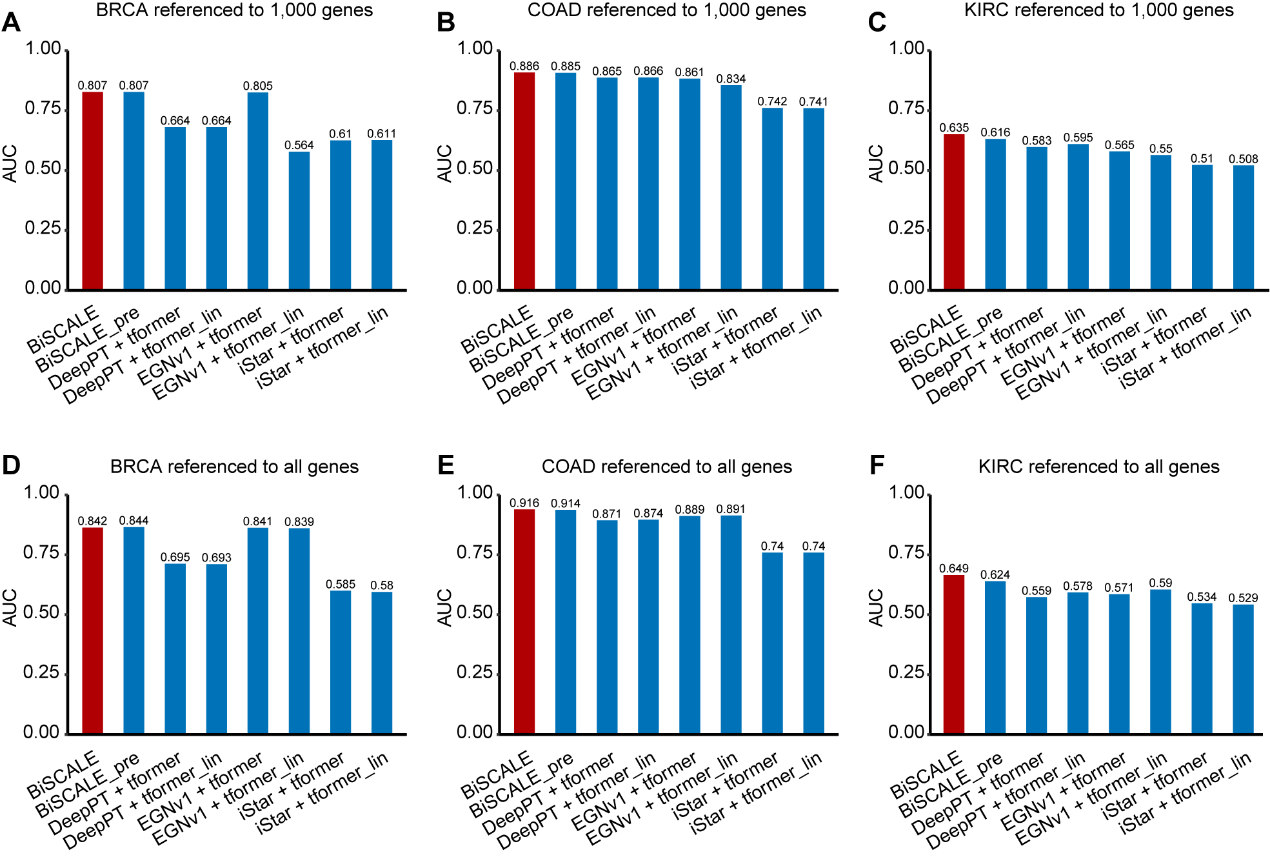


**Figure S5.** **Comparative assessment of expression similarity for spot-level and bulk-level predictions against ground truth.** (A–C) AUC values quantifying prediction accuracy for the 1,000 target genes. (D–F) AUC values quantifying prediction accuracy for all expressed genes.


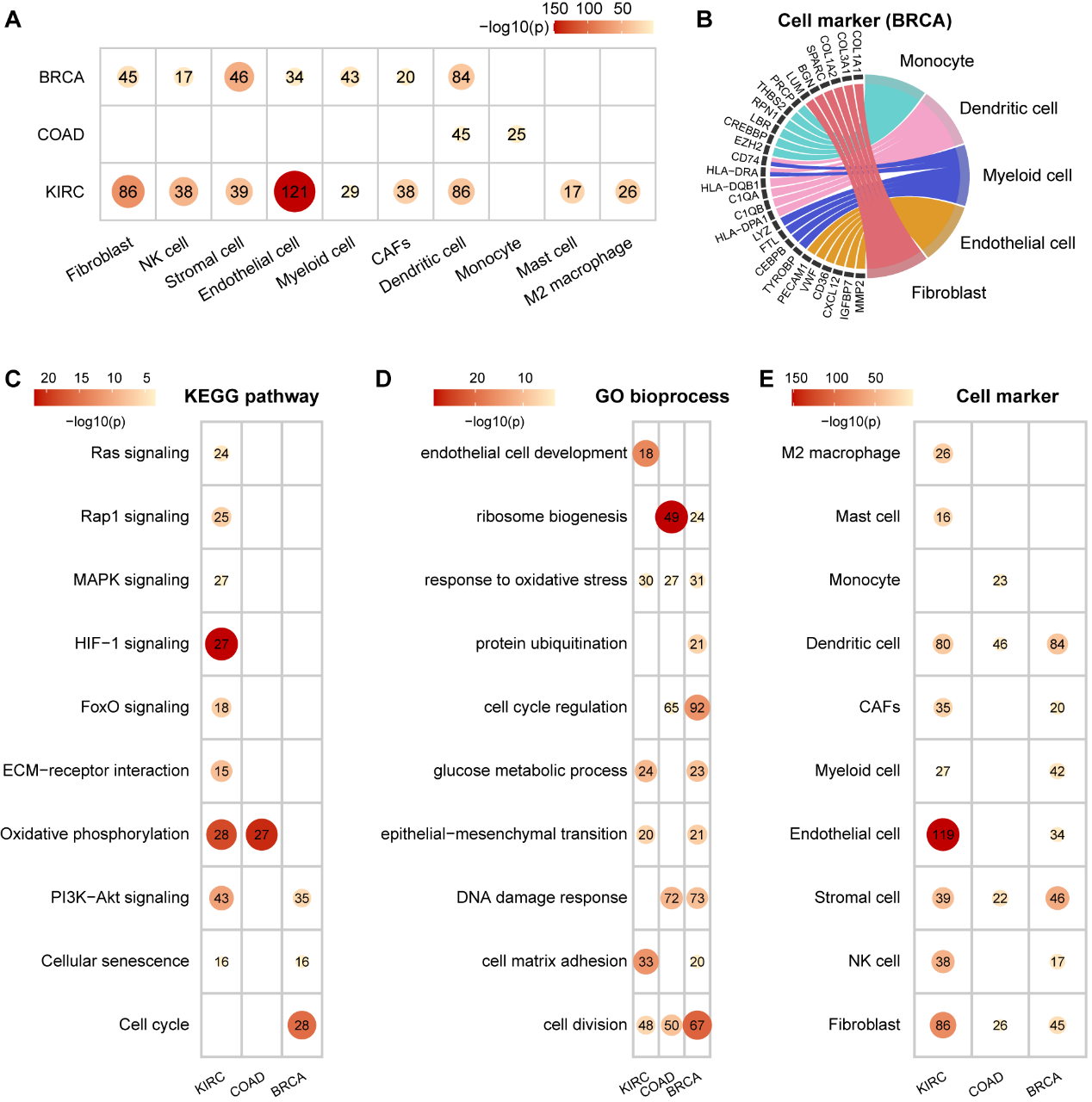


**Figure S6. Pathway enrichment analyses of BiSCALE-predicted gene expression.** (A) Heatmap showing the significance of cell marker enrichment for well-predicted genes at the bulk level. Circle color and size represent the negative log-transformed p-values, and integers indicate gene counts in each pathway. (B) Circos plots showing cell marker enrichment with well-predicted genes in BRCA. (C-E) Pathway enrichment analysis of well-predicted genes in the independent CPTAC cohort.


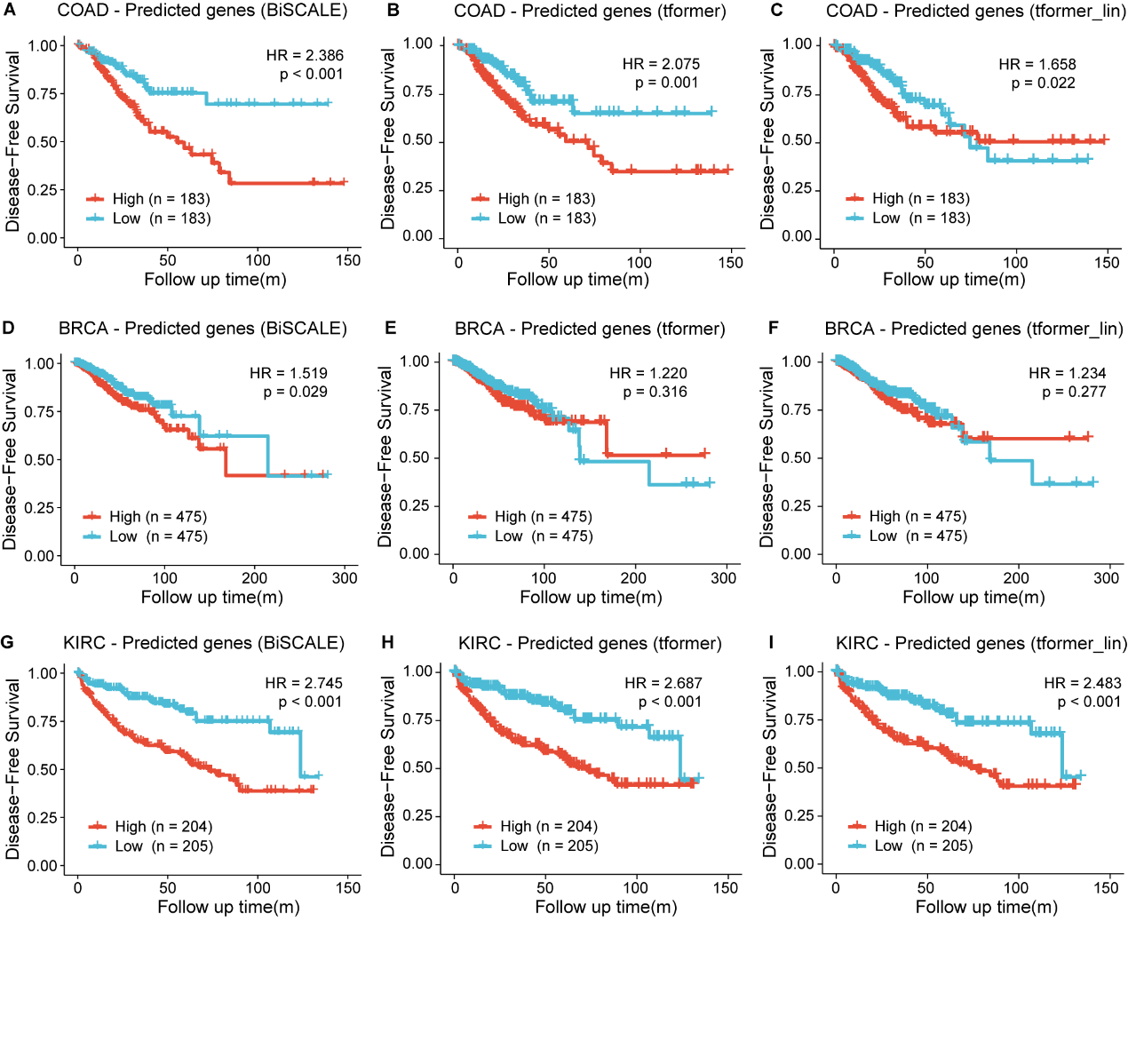


**Figure S7. Clinical phenotype stratification using WSI-predicted bulk gene expression.** Kaplan–Meier curves compare recurrence-free survival between high- and low-risk groups stratified by Cox risk scores computed from model-predicted expression. (A–C) COAD results using predictions from (A) BiSCALE, (B) tformer, and (C) tformer_lin. (D–F) BRCA results using three methods. (G–I) KIRC results using three methods.


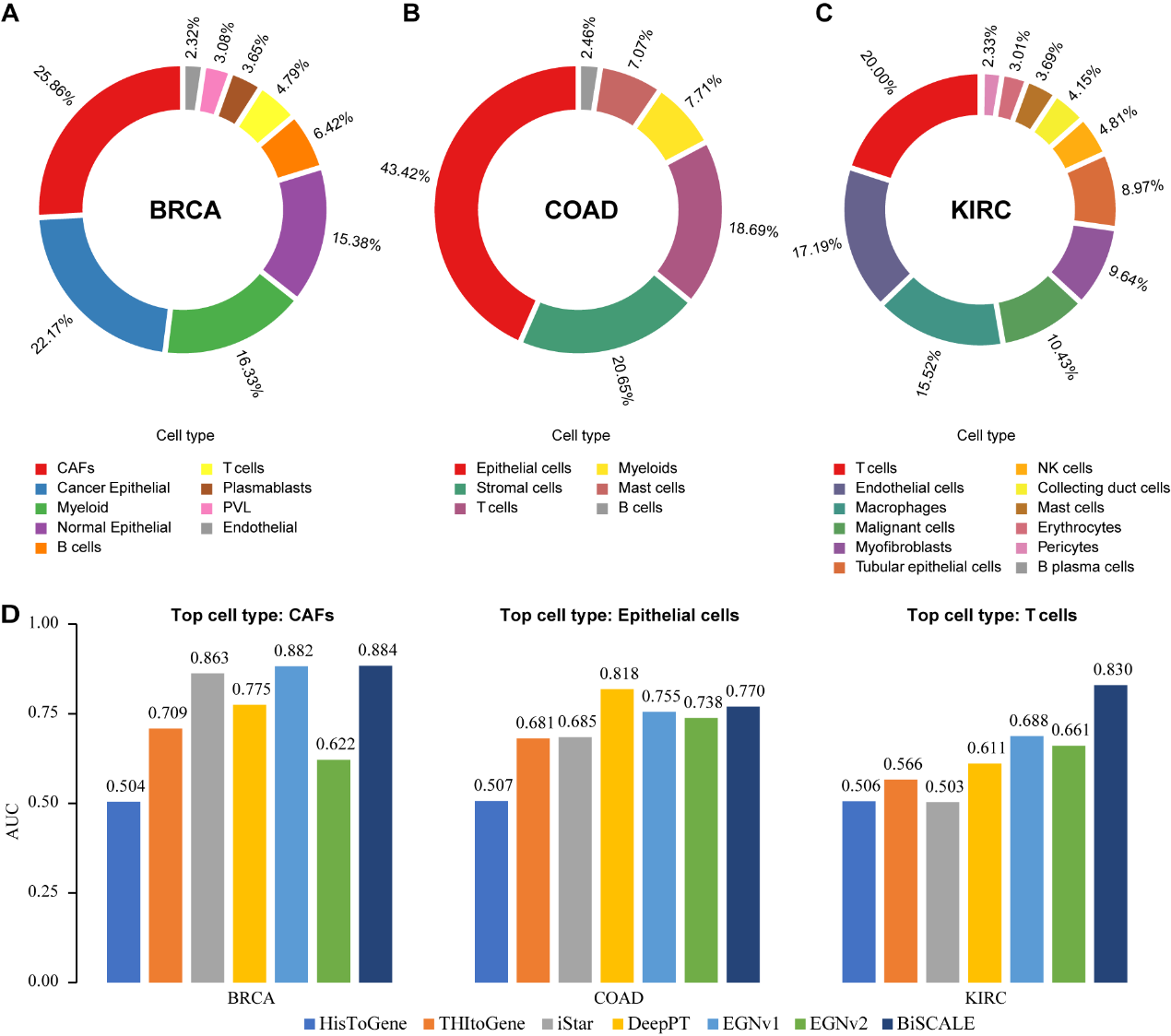


**Figure S8. Cell type proportions and dominant cell-type annotation in spatial transcriptomics spots.** (A–C) Cell type proportions per spot for (A) BRCA, (B) COAD, and (C) KIRC inferred from BiSCALE-predicted expression using CARD. (D) Top-1 dominant cell-type annotation performance measured by AUC, comparing BiSCALE with whole-image and spot-conditioned baselines across the three cancer types.


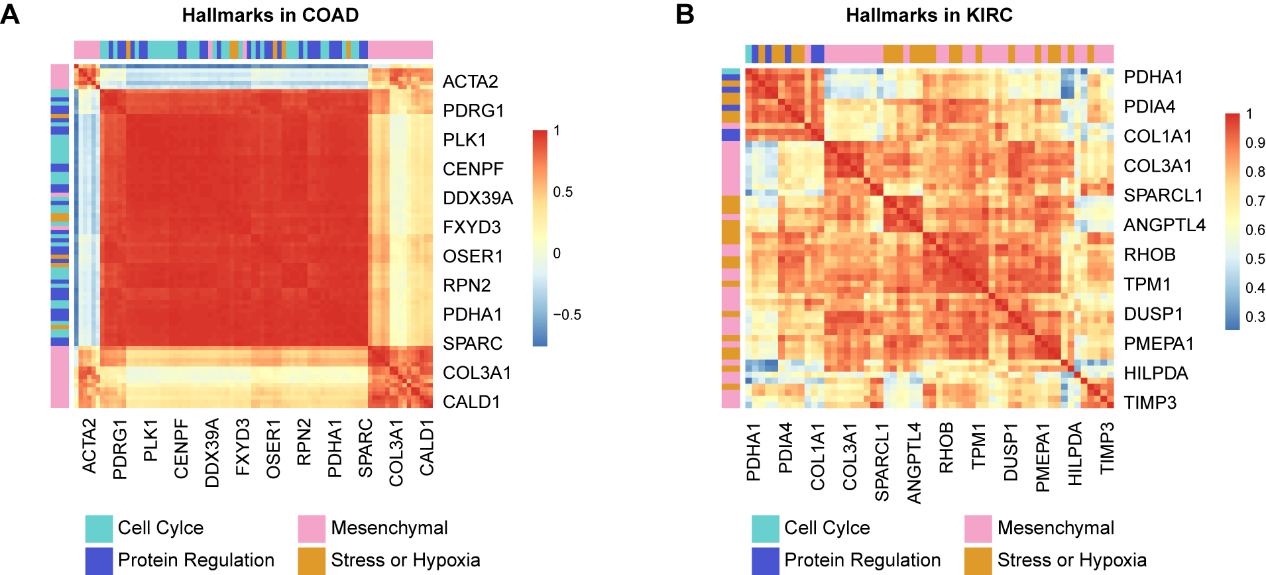


**Figure S9. BiSCALE performance in spatial co-expression analysis.** Heatmap showing the Pearson correlation coefficients for functionally related genes from established meta-programs of transcriptional intratumor heterogeneity in (A) COAD and (B) KIRC.


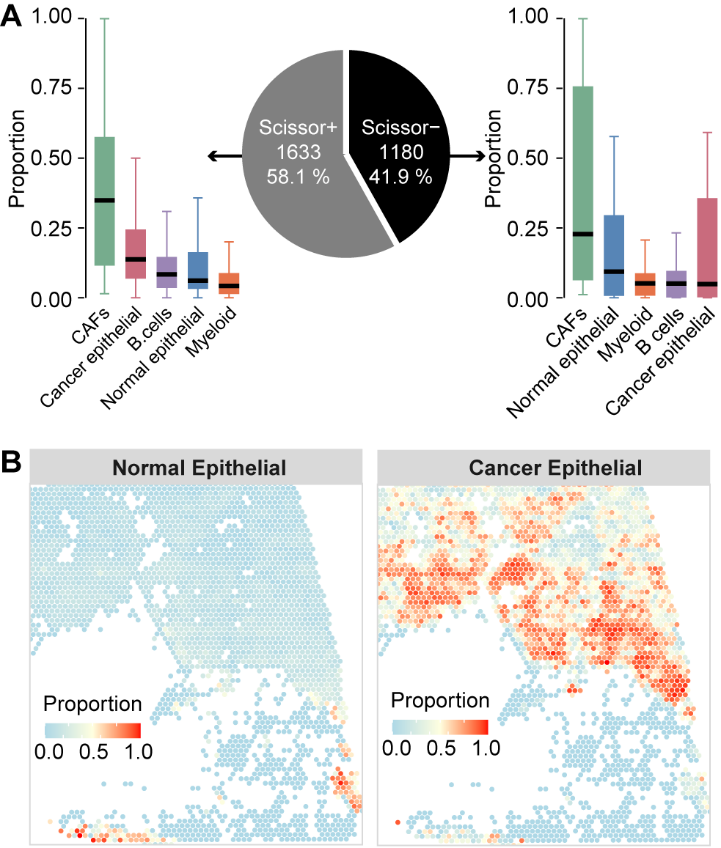


**Figure S10.** (D) The pie chart of the Scissor-selected cells with the corresponding bar plots showing the cell type-specific proportions based on BiSCALE-predicted expression. (E) Spatial maps of cancer epithelial and normal epithelial cell distribution based on BiSCALE-predicted expression.

**Supplementary Table S1.** Number of samples with paired Whole Slide Images and RNA-seq data.

| Type | Project | number patients |
| --- | --- | --- |
| Training | TCGA-BRCA | 1059 |
|  | TCGA-COAD | 447 |
|  | TCGA-KIRC | 603 |
| Testing | CPTAC-BRCA | 106 |
|  | CPTAC-COAD | 103 |
|  | CPTAC-KIRC | 286 |
| Sum | | 2604 |

**Supplementary Table S2.** Number of samples in spatial transcriptome data

| Type | Cancer Type | Number samples | Number spots | Links |
| --- | --- | --- | --- | --- |
| Training | COAD | 27 | 40,954 | <https://data.humantumoratlas.org/publications/hta11_2023_cell_cody-n-heiser> |
|  | BRCA | 27 | 75,739 | <https://data.humantumoratlas.org/publications/hta12_2024_nature-cancer_michael-d-iglesia> |
|  | KIRC | 8 | 24,343 | <https://data.humantumoratlas.org/publications/hta12_2024_nature_chia-kuei-mo> |
| Testing | COAD | 17 | 47,547 | <https://data.humantumoratlas.org/publications/hta12_2024_nature_chia-kuei-mo> |
|  | BRCA | 15 | 32,350 | <https://data.humantumoratlas.org/publications/hta12_2024_nature_chia-kuei-mo> |
| Sum | | 94 | 220,933 |  |

**Supplementary Table S3.** Median correlation coefficient (test sets)

|  | Methods | BRCA | COAD | KIRC |
| --- | --- | --- | --- | --- |
| Bulk-level | tformer | 0.405 | 0.383 | 0.442 |
|  | tformer_lin | 0.434 | 0.398 | 0.443 |
|  | BiSCALE_pre | 0.457 | 0.415 | 0.487 |
|  | BiSCALE | **0.470** | **0.421** | **0.491** |
| Spot-level | HisToGene | 0.001 | 0.002 | 0.001 |
|  | THItoGene | 0.128 | 0.177 | 0.049 |
|  | iStar | 0.191 | 0.179 | -0.002 |
|  | DeepPT | 0.152 | **0.267** | 0.080 |
|  | EGNv1 | 0.201 | 0.221 | 0.099 |
|  | EGNv2 | 0.102 | 0.210 | 0.092 |
|  | BiSCALE_pre | 0.202 | 0.230 | 0.146 |
|  | BiSCALE | **0.202** | 0.231 | **0.147** |

Note: The best-performing results are highlighted in bold.

**Supplementary Table S4.** Number of well-predicted genes (test sets)

|  | Methods | BRCA | COAD | KIRC |
| --- | --- | --- | --- | --- |
| Bulk-level | tformer | 513 | 449 | 667 |
|  | tformer_lin | 628 | 490 | 642 |
|  | BiSCALE_pre | 732 | 534 | 766 |
|  | BiSCALE | **770** | **564** | **777** |
| Spot-level | HisToGene | 0 | 0 | 0 |
|  | THItoGene | 195 | 328 | 6 |
|  | iStar | 470 | 399 | 0 |
|  | DeepPT | 206 | **863** | 0 |
|  | EGNv1 | 511 | 650 | 4 |
|  | EGNv2 | 7 | 631 | 0 |
|  | BiSCALE_pre | **518** | 650 | **121** |
|  | BiSCALE | **518** | 642 | 108 |

Note: The best-performing results are highlighted in bold.

**Supplementary Table S5.** P-values for overall differences in gene correlation between BiSCALE and other methods (test sets)

|  | Methods | BRCA | COAD | KIRC |
| --- | --- | --- | --- | --- |
| Bulk-level | tformer | < 0.001 | < 0.001 | < 0.001 |
|  | tformer_lin | < 0.001 | < 0.001 | < 0.001 |
|  | BiSCALE_pre | 0.004 | 0.075 | 0.279 |
| Spot-level | HisToGene | < 0.001 | < 0.001 | < 0.001 |
|  | THItoGene | < 0.001 | < 0.001 | < 0.001 |
|  | iStar | 0.013 | < 0.001 | < 0.001 |
|  | DeepPT | < 0.001 | < 0.001 | < 0.001 |
|  | EGNv1 | < 0.001 | 0.809 | < 0.001 |
|  | EGNv2 | < 0.001 | < 0.001 | < 0.001 |
|  | BiSCALE_pre | 0.960 | 0.763 | 0.972 |

**Supplementary Table S6.** Cross-cancer performance of bulk gene expression prediction on 16 TCGA cohorts.

| Project | number patients | corrlation (tformer) | corrlation (tformer_lin) | corrlation (BiSCALE) |
| --- | --- | --- | --- | --- |
| TCGA-BLCA | 386 | 0.47 | 0.374 | **0.561** |
| TCGA-BRCA | 1059 | 0.405 | 0.434 | **0.47** |
| TCGA-COAD | 447 | 0.383 | 0.398 | **0.421** |
| TCGA-GBM | 385 | 0.104 | 0.159 | **0.218** |
| TCGA-HNSC | 441 | 0.443 | 0.47 | **0.548** |
| TCGA-KIRC | 603 | 0.442 | 0.443 | **0.491** |
| TCGA-KIRP | 270 | 0.456 | 0.557 | **0.593** |
| TCGA-LIHC | 365 | 0.574 | 0.6 | **0.638** |
| TCGA-LUAD | 537 | 0.42 | 0.442 | **0.45** |
| TCGA-LUSC | 512 | 0.31 | 0.333 | **0.35** |
| TCGA-PAAD | 183 | **-0.005** | -0.005 | -0.005 |
| TCGA-PRAD | 393 | 0.289 | **0.301** | 0.202 |
| TCGA-SKCM | 282 | **0.155** | -0.203 | -0.081 |
| TCGA-STAD | 360 | 0.405 | 0.422 | **0.523** |
| TCGA-THCA | 498 | 0.563 | 0.542 | **0.639** |
| TCGA-UCEC | 504 | 0.305 | 0.364 | **0.38** |

Note: The best-performing results are highlighted in bold.

**Supplementary Table S7.** Median correlation coefficient (independent test sets)

|  | Methods | BRCA | COAD | KIRC |
| --- | --- | --- | --- | --- |
| Bulk-level | tformer | 0.365 | 0.102 | 0.235 |
|  | tformer_lin | 0.416 | 0.106 | 0.236 |
|  | BiSCALE_pre | 0.434 | 0.184 | 0.276 |
|  | BiSCALE | **0.435** | **0.203** | **0.284** |
| Spot-level | HisToGene | -0.001 | -0.002 | - |
|  | THItoGene | 0.262 | 0.030 | - |
|  | iStar | 0.149 | 0.079 | - |
|  | DeepPT | 0.117 | 0.130 | - |
|  | EGNv1 | 0.088 | 0.172 | - |
|  | EGNv2 | 0.063 | 0.189 | - |
|  | BiSCALE_pre | 0.282 | **0.212** | - |
|  | BiSCALE | **0.283** | 0.206 | - |

Note: The best-performing results are highlighted in bold.

**Supplementary Table S8.** P-values for overall differences in gene correlation between BiSCALE and other methods (independent test sets)

|  | Methods | BRCA | COAD | KIRC |
| --- | --- | --- | --- | --- |
| Bulk-level | tformer | < 0.001 | < 0.001 | < 0.001 |
|  | tformer_lin | 0.012 | < 0.001 | < 0.001 |
|  | BiSCALE_pre | 0.868 | 0.006 | 0.360 |
| Spot-level | HisToGene | < 0.001 | < 0.001 | - |
|  | THItoGene | < 0.001 | < 0.001 | - |
|  | iStar | < 0.001 | < 0.001 | - |
|  | DeepPT | < 0.001 | < 0.001 | - |
|  | EGNv1 | < 0.001 | < 0.001 | - |
|  | EGNv2 | < 0.001 | < 0.001 | - |
|  | BiSCALE_pre | 0.003 | 0.697 | - |

**Supplementary Table S9.** Mean correlation coefficient between actual and predicted pathway scores for KEGG pathway.

|  | Methods | BRCA | COAD | KIRC |
| --- | --- | --- | --- | --- |
| Bulk-level | tformer | 0.490 | 0.323 | 0.272 |
|  | tformer_lin | 0.506 | 0.329 | 0.272 |
|  | BiSCALE | **0.524** | **0.337** | **0.285** |
| Spot-level | HisToGene | 0.002 | 0.006 | 0.004 |
|  | THItoGene | 0.319 | 0.563 | 0.280 |
|  | iStar | 0.388 | 0.566 | 0.001 |
|  | DeepPT | 0.349 | **0.677** | 0.394 |
|  | EGNv1 | 0.397 | 0.625 | 0.444 |
|  | EGNv2 | 0.280 | 0.611 | 0.427 |
|  | BiSCALE | **0.398** | 0.637 | **0.536** |

Note: The best-performing results are highlighted in bold.

**Supplementary Table S10.** Mean correlation coefficient between actual and predicted pathway scores for GO bioprocess.

|  | Methods | BRCA | COAD | KIRC |
| --- | --- | --- | --- | --- |
| Bulk-level | tformer | 0.448 | 0.293 | 0.278 |
|  | tformer_lin | 0.491 | 0.308 | 0.279 |
|  | BiSCALE | **0.540** | **0.329** | **0.316** |
| Spot-level | HisToGene | 0.001 | 0.003 | 0.003 |
|  | THItoGene | 0.070 | 0.205 | 0.121 |
|  | iStar | 0.138 | 0.209 | 0.001 |
|  | DeepPT | 0.099 | **0.344** | 0.145 |
|  | EGNv1 | 0.146 | 0.280 | 0.261 |
|  | EGNv2 | 0.032 | 0.263 | 0.221 |
|  | BiSCALE | **0.147** | 0.295 | **0.476** |

Note: The best-performing results are highlighted in bold.

**Supplementary Table S11.** Mean correlation coefficient between actual and predicted pathway scores for Cell marker.

|  | Methods | BRCA | COAD | KIRC |
| --- | --- | --- | --- | --- |
| Bulk-level | tformer | 0.478 | 0.329 | 0.329 |
|  | tformer_lin | 0.513 | 0.342 | 0.330 |
|  | BiSCALE | **0.553** | **0.360** | **0.364** |
| Spot-level | HisToGene | 0.002 | 0.004 | 0.003 |
|  | THItoGene | 0.192 | 0.358 | 0.000 |
|  | iStar | 0.312 | 0.363 | 0.001 |
|  | DeepPT | 0.244 | **0.535** | 0.169 |
|  | EGNv1 | 0.328 | 0.454 | 0.242 |
|  | EGNv2 | 0.124 | 0.432 | 0.217 |
|  | BiSCALE | **0.329** | 0.473 | **0.378** |

Note: The best-performing results are highlighted in bold.

**Supplementary Table S12.** Significant enrichment of gene sets in KEGG pathways.

| Term | Type | Gene count | P-values |
| --- | --- | --- | --- |
| AMPK signaling pathway | BRCA | 15 | 7.87E-03 |
| Alzheimer disease | BRCA | 40 | 8.67E-04 |
| Amyotrophic lateral sclerosis | BRCA | 40 | 2.99E-04 |
| Cell cycle | BRCA | 27 | 1.09E-06 |
| Cellular senescence | BRCA | 18 | 7.88E-03 |
| Epstein-Barr virus infection | BRCA | 26 | 3.17E-04 |
| Focal adhesion | BRCA | 21 | 1.28E-02 |
| Herpes simplex virus 1 infection | BRCA | 20 | 8.37E-03 |
| Human T-cell leukemia virus 1 infection | BRCA | 26 | 1.32E-03 |
| Huntington disease | BRCA | 35 | 3.09E-04 |
| Oocyte meiosis | BRCA | 18 | 1.98E-03 |
| PI3K-Akt signaling pathway | BRCA | 34 | 8.94E-03 |
| Parkinson disease | BRCA | 31 | 4.98E-04 |
| Phagosome | BRCA | 18 | 6.05E-03 |
| Prion disease | BRCA | 33 | 1.66E-04 |
| Spinocerebellar ataxia | BRCA | 18 | 3.17E-03 |
| Spliceosome | BRCA | 20 | 2.41E-02 |
| Systemic lupus erythematosus | BRCA | 15 | 3.00E-02 |
| Thermogenesis | BRCA | 23 | 1.77E-02 |
| Tuberculosis | BRCA | 19 | 1.50E-02 |
| Alzheimer disease | COAD | 50 | 3.56E-09 |
| Amyotrophic lateral sclerosis | COAD | 52 | 5.63E-11 |
| Cell cycle | COAD | 15 | 2.10E-02 |
| Chemical carcinogenesis | COAD | 31 | 1.11E-06 |
| Diabetic cardiomyopathy | COAD | 32 | 3.18E-08 |
| Huntington disease | COAD | 46 | 1.21E-10 |
| Metabolic pathways | COAD | 107 | 2.33E-03 |
| Non-alcoholic fatty liver disease | COAD | 24 | 2.55E-06 |
| Nucleocytoplasmic transport | COAD | 22 | 3.93E-08 |
| Oxidative phosphorylation | COAD | 30 | 1.53E-11 |
| Parkinson disease | COAD | 44 | 1.28E-11 |
| Pathways of neurodegeneration | COAD | 54 | 9.62E-08 |
| Prion disease | COAD | 40 | 4.66E-09 |
| Ribosome biogenesis in eukaryotes | COAD | 19 | 2.59E-04 |
| Spliceosome | COAD | 31 | 7.84E-08 |
| Thermogenesis | COAD | 37 | 1.77E-09 |
| Tight junction | COAD | 15 | 3.73E-02 |
| mRNA surveillance pathway | COAD | 17 | 2.71E-05 |
| AGE-RAGE signaling pathway in diabetic complications | KIRC | 18 | 6.27E-05 |
| Alzheimer disease | KIRC | 46 | 3.12E-05 |
| Carbon metabolism | KIRC | 21 | 1.22E-05 |
| Cardiac muscle contraction | KIRC | 17 | 2.95E-05 |
| Central carbon metabolism in cancer | KIRC | 15 | 3.31E-05 |
| Chemical carcinogenesis | KIRC | 41 | 9.30E-10 |
| Cytoskeleton in muscle cells | KIRC | 40 | 6.10E-09 |
| Diabetic cardiomyopathy | KIRC | 40 | 1.34E-10 |
| EGFR tyrosine kinase inhibitor resistance | KIRC | 18 | 2.11E-06 |
| Focal adhesion | KIRC | 37 | 4.25E-09 |
| HIF-1 signaling pathway | KIRC | 28 | 1.48E-10 |
| Human papillomavirus infection | KIRC | 42 | 1.48E-05 |
| Leukocyte transendothelial migration | KIRC | 20 | 4.08E-05 |
| Metabolic pathways | KIRC | 143 | 1.56E-06 |
| Non-alcoholic fatty liver disease | KIRC | 29 | 1.73E-07 |
| Oxidative phosphorylation | KIRC | 32 | 6.61E-11 |
| PI3K-Akt signaling pathway | KIRC | 44 | 2.53E-05 |
| Parkinson disease | KIRC | 45 | 1.58E-09 |
| Pathways in cancer | KIRC | 66 | 1.03E-07 |
| Prion disease | KIRC | 36 | 2.92E-05 |

Note: Only the top 20 most significant gene sets for each cancer type are displayed.

**Supplementary Table S13.** Significant enrichment of gene sets in Gene Ontology

| Term | Type | Gene_count | P-values |
| --- | --- | --- | --- |
| GOBP_CELLULAR_RESPONSE_TO_STRESS | BRCA | 156 | 1.72E-09 |
| GOBP_CELL_CYCLE | BRCA | 136 | 1.13E-08 |
| GOBP_CELL_CYCLE_PROCESS | BRCA | 112 | 3.24E-08 |
| GOBP_CELL_DIVISION | BRCA | 69 | 3.64E-10 |
| GOBP_CHROMOSOME_ORGANIZATION | BRCA | 65 | 2.12E-09 |
| GOBP_DNA_BIOSYNTHETIC_PROCESS | BRCA | 27 | 2.77E-08 |
| GOBP_DNA_METABOLIC_PROCESS | BRCA | 97 | 1.99E-10 |
| GOBP_DNA_REPLICATION | BRCA | 35 | 2.65E-07 |
| GOBP_MITOTIC_CELL_CYCLE | BRCA | 96 | 6.93E-12 |
| GOBP_MITOTIC_CELL_CYCLE_PHASE_TRANSITION | BRCA | 51 | 4.09E-08 |
| GOBP_MITOTIC_CELL_CYCLE_PROCESS | BRCA | 86 | 2.42E-12 |
| GOBP_POSITIVE_REGULATION_OF_CHROMOSOME_ORGANIZATION | BRCA | 22 | 4.94E-08 |
| GOBP_POSITIVE_REGULATION_OF_DNA_BIOSYNTHETIC_PROCESS | BRCA | 17 | 1.77E-10 |
| GOBP_POSITIVE_REGULATION_OF_DNA_METABOLIC_PROCESS | BRCA | 39 | 2.74E-08 |
| GOBP_REGULATION_OF_CELL_CYCLE | BRCA | 96 | 6.97E-08 |
| GOBP_REGULATION_OF_CHROMOSOME_ORGANIZATION | BRCA | 35 | 2.84E-08 |
| GOBP_REGULATION_OF_DNA_BIOSYNTHETIC_PROCESS | BRCA | 20 | 2.01E-08 |
| GOBP_REGULATION_OF_DNA_METABOLIC_PROCESS | BRCA | 60 | 3.97E-10 |
| GOBP_RESPONSE_TO_UV | BRCA | 24 | 4.06E-07 |
| GOBP_SISTER_CHROMATID_SEGREGATION | BRCA | 32 | 2.04E-07 |
| GOBP_AEROBIC_RESPIRATION | COAD | 37 | 1.19E-12 |
| GOBP_ATP_SYNTHESIS_COUPLED_ELECTRON_TRANSPORT | COAD | 23 | 7.93E-10 |
| GOBP_CELLULAR_RESPIRATION | COAD | 40 | 4.65E-12 |
| GOBP_DNA_METABOLIC_PROCESS | COAD | 90 | 3.53E-09 |
| GOBP_ESTABLISHMENT_OF_RNA_LOCALIZATION | COAD | 30 | 3.39E-10 |
| GOBP_GENERATION_OF_PRECURSOR_METABOLITES_AND_ENERGY | COAD | 53 | 5.08E-09 |
| GOBP_MITOTIC_SISTER_CHROMATID_SEGREGATION | COAD | 31 | 3.21E-09 |
| GOBP_MRNA_METABOLIC_PROCESS | COAD | 97 | 2.03E-14 |
| GOBP_MRNA_PROCESSING | COAD | 75 | 3.73E-17 |
| GOBP_MRNA_TRANSPORT | COAD | 27 | 1.32E-10 |
| GOBP_OXIDATIVE_PHOSPHORYLATION | COAD | 34 | 7.95E-14 |
| GOBP_PROTON_MOTIVE_FORCE_DRIVEN_ATP_SYNTHESIS | COAD | 20 | 1.35E-09 |
| GOBP_RIBONUCLEOPROTEIN_COMPLEX_BIOGENESIS | COAD | 78 | 5.18E-20 |
| GOBP_RIBOSOME_BIOGENESIS | COAD | 55 | 2.98E-16 |
| GOBP_RNA_LOCALIZATION | COAD | 42 | 5.13E-15 |
| GOBP_RNA_PROCESSING | COAD | 133 | 1.35E-14 |
| GOBP_RNA_SPLICING | COAD | 70 | 3.06E-16 |
| GOBP_RNA_SPLICING_VIA_TRANSESTERIFICATION_REACTIONS | COAD | 56 | 1.92E-15 |
| GOBP_RRNA_METABOLIC_PROCESS | COAD | 50 | 4.09E-17 |
| GOBP_RRNA_PROCESSING | COAD | 42 | 1.44E-14 |
| GOBP_ANATOMICAL_STRUCTURE_FORMATION_INVOLVED_IN_MORPHOGENESIS | KIRC | 137 | 2.09E-16 |
| GOBP_BLOOD_VESSEL_MORPHOGENESIS | KIRC | 106 | 7.97E-24 |
| GOBP_CELLULAR_RESPONSE_TO_ENDOGENOUS_STIMULUS | KIRC | 129 | 1.11E-13 |
| GOBP_CELL_JUNCTION_ORGANIZATION | KIRC | 96 | 2.01E-13 |
| GOBP_CELL_MOTILITY | KIRC | 171 | 5.22E-14 |
| GOBP_CELL_SURFACE_RECEPTOR_PROTEIN_TYROSINE_KINASE_SIGNALING_PATHWAY | KIRC | 82 | 6.53E-14 |
| GOBP_CIRCULATORY_SYSTEM_DEVELOPMENT | KIRC | 154 | 1.75E-26 |
| GOBP_ENERGY_DERIVATION_BY_OXIDATION_OF_ORGANIC_COMPOUNDS | KIRC | 54 | 1.50E-13 |
| GOBP_ENZYME_LINKED_RECEPTOR_PROTEIN_SIGNALING_PATHWAY | KIRC | 115 | 5.67E-15 |
| GOBP_GENERATION_OF_PRECURSOR_METABOLITES_AND_ENERGY | KIRC | 68 | 8.17E-15 |
| GOBP_GLOMERULUS_DEVELOPMENT | KIRC | 26 | 1.85E-16 |
| GOBP_NEPHRON_DEVELOPMENT | KIRC | 37 | 1.45E-15 |
| GOBP_POSITIVE_REGULATION_OF_LOCOMOTION | KIRC | 80 | 1.56E-13 |
| GOBP_REGULATION_OF_LOCOMOTION | KIRC | 121 | 4.12E-14 |
| GOBP_RENAL_SYSTEM_DEVELOPMENT | KIRC | 57 | 1.13E-16 |
| GOBP_RESPONSE_TO_GROWTH_FACTOR | KIRC | 90 | 1.11E-13 |
| GOBP_SMALL_MOLECULE_METABOLIC_PROCESS | KIRC | 168 | 4.72E-16 |
| GOBP_TUBE_DEVELOPMENT | KIRC | 139 | 8.84E-21 |
| GOBP_TUBE_MORPHOGENESIS | KIRC | 124 | 8.95E-22 |
| GOBP_VASCULATURE_DEVELOPMENT | KIRC | 118 | 1.78E-24 |

Note: Only the top 20 most significant gene sets for each cancer type are displayed.

**Supplementary Table S14.** Significant enrichment of gene sets in Cell marker sets.

| Term | Type | Gene_count | P-values |
| --- | --- | --- | --- |
| BUSSLINGER_DUODENAL_DIFFERENTIATING_STEM_CELLS | BRCA | 62 | 8.00E-21 |
| BUSSLINGER_GASTRIC_ISTHMUS_CELLS | BRCA | 76 | 4.96E-21 |
| FAN_EMBRYONIC_CTX_MICROGLIA_1 | BRCA | 46 | 3.55E-23 |
| FAN_EMBRYONIC_CTX_NSC_2 | BRCA | 55 | 2.26E-22 |
| FAN_OVARY_CL1_GPRC5A_TNFRS12A_HIGH_SELECTABLE_FOLLICLE_STROMAL_CELL | BRCA | 76 | 6.65E-26 |
| FAN_OVARY_CL6_PUTATIVE_EARLY_ATRETIC_FOLLICLE_THECAL_CELL_2 | BRCA | 78 | 2.33E-34 |
| HAY_BONE_MARROW_ERYTHROBLAST | BRCA | 193 | 1.14E-47 |
| HE_LIM_SUN_FETAL_LUNG_C5_LARGE_PRE_B_CELL | BRCA | 179 | 4.07E-34 |
| HE_LIM_SUN_FETAL_LUNG_C5_PRO_B_CELL | BRCA | 81 | 7.77E-23 |
| JONES_OVARY_STROMAL | BRCA | 46 | 3.04E-24 |
| MANNO_MIDBRAIN_NEUROTYPES_HPROGFPM | BRCA | 68 | 5.88E-24 |
| RUBENSTEIN_SKELETAL_MUSCLE_FAP_CELLS | BRCA | 50 | 8.80E-24 |
| SU_HO_CONV_CENT_CHONDROSARCOMA_C7_PROLIFERATIVE | BRCA | 62 | 8.00E-29 |
| SU_HO_FOETAL_FEMUR_C3_PROLIFERATING_CHONDROCYTE | BRCA | 104 | 1.33E-34 |
| TRAVAGLINI_LUNG_PROLIFERATING_BASAL_CELL | BRCA | 167 | 3.79E-51 |
| TRAVAGLINI_LUNG_PROLIFERATING_MACROPHAGE_CELL | BRCA | 188 | 3.69E-58 |
| TRAVAGLINI_LUNG_PROLIFERATING_NK_T_CELL | BRCA | 43 | 6.24E-26 |
| ZHONG_PFC_C1_MICROGLIA | BRCA | 72 | 2.75E-32 |
| ZHONG_PFC_C1_OPC | BRCA | 56 | 3.00E-23 |
| ZHONG_PFC_MAJOR_TYPES_NPCS | BRCA | 49 | 1.60E-28 |
| BUSSLINGER_DUODENAL_DIFFERENTIATING_STEM_CELLS | COAD | 60 | 1.23E-20 |
| BUSSLINGER_DUODENAL_STEM_CELLS | COAD | 50 | 3.23E-14 |
| BUSSLINGER_GASTRIC_ISTHMUS_CELLS | COAD | 76 | 1.79E-22 |
| BUSSLINGER_GASTRIC_PARIETAL_CELLS | COAD | 41 | 6.61E-13 |
| FAN_OVARY_CL10_PUTATIVE_EARLY_ATRESIA_GRANULOSA_CELL | COAD | 66 | 5.32E-27 |
| FAN_OVARY_CL15_SMALL_ANTRAL_FOLLICLE_GRANULOSA_CELL | COAD | 71 | 1.07E-11 |
| FAN_OVARY_CL8_MATURE_CUMULUS_GRANULOSA_CELL_2 | COAD | 126 | 3.41E-44 |
| GAUTAM_EYE_IRIS_CILIARY_BODY_MELANOCYTES | COAD | 38 | 3.39E-11 |
| HAY_BONE_MARROW_ERYTHROBLAST | COAD | 161 | 1.05E-32 |
| HE_LIM_SUN_FETAL_LUNG_C2_CYCLING_DC_CELL | COAD | 131 | 4.17E-14 |
| HE_LIM_SUN_FETAL_LUNG_C3_EARLY_CAP_CELL | COAD | 73 | 2.37E-11 |
| HE_LIM_SUN_FETAL_LUNG_C5_LARGE_PRE_B_CELL | COAD | 142 | 1.52E-19 |
| MENON_FETAL_KIDNEY_7_LOOPOF_HENLE_CELLS_DISTAL | COAD | 42 | 5.70E-12 |
| MENON_FETAL_KIDNEY_8_CONNECTING_TUBULE_CELLS | COAD | 45 | 8.15E-14 |
| RUBENSTEIN_SKELETAL_MUSCLE_SMOOTH_MUSCLE_CELLS | COAD | 57 | 3.81E-12 |
| SU_HO_CONV_CENT_CHONDROSARCOMA_C5_CHON2 | COAD | 45 | 1.33E-13 |
| SU_HO_CONV_CENT_CHONDROSARCOMA_C7_PROLIFERATIVE | COAD | 43 | 5.30E-15 |
| TRAVAGLINI_LUNG_PROLIFERATING_BASAL_CELL | COAD | 140 | 1.52E-36 |
| TRAVAGLINI_LUNG_PROXIMAL_BASAL_CELL | COAD | 80 | 3.43E-16 |
| TRAVAGLINI_LUNG_PROXIMAL_CILIATED_CELL | COAD | 192 | 5.29E-30 |
| AIZARANI_LIVER_C10_MVECS_1 | KIRC | 113 | 1.71E-75 |
| AIZARANI_LIVER_C13_LSECS_2 | KIRC | 90 | 4.47E-48 |
| AIZARANI_LIVER_C20_LSECS_3 | KIRC | 84 | 1.70E-41 |
| AIZARANI_LIVER_C29_MVECS_2 | KIRC | 116 | 3.13E-69 |
| AIZARANI_LIVER_C9_LSECS_1 | KIRC | 88 | 4.90E-44 |
| CUI_DEVELOPING_HEART_C4_ENDOTHELIAL_CELL | KIRC | 61 | 1.51E-39 |
| DESCARTES_FETAL_CEREBELLUM_VASCULAR_ENDOTHELIAL_CELLS | KIRC | 120 | 5.45E-40 |
| FAN_EMBRYONIC_CTX_BIG_GROUPS_BRAIN_ENDOTHELIAL | KIRC | 127 | 6.02E-70 |
| FAN_EMBRYONIC_CTX_BRAIN_ENDOTHELIAL_1 | KIRC | 125 | 6.02E-61 |
| FAN_EMBRYONIC_CTX_BRAIN_ENDOTHELIAL_2 | KIRC | 100 | 6.64E-57 |
| FAN_OVARY_CL14_MATURE_SMOOTH_MUSCLE_CELL | KIRC | 95 | 1.28E-47 |
| GAO_LARGE_INTESTINE_ADULT_CJ_IMMUNE_CELLS | KIRC | 116 | 3.78E-47 |
| JONES_OVARY_ENDOTHELIAL | KIRC | 73 | 6.63E-43 |
| JONES_OVARY_PERICYTE | KIRC | 73 | 1.05E-46 |
| MANNO_MIDBRAIN_NEUROTYPES_HENDO | KIRC | 197 | 7.14E-80 |
| MANNO_MIDBRAIN_NEUROTYPES_HPERIC | KIRC | 165 | 3.77E-61 |
| MURARO_PANCREAS_DUCTAL_CELL | KIRC | 238 | 1.67E-67 |
| MURARO_PANCREAS_ENDOTHELIAL_CELL | KIRC | 121 | 4.61E-66 |
| MURARO_PANCREAS_MESENCHYMAL_STROMAL_CELL | KIRC | 126 | 2.64E-40 |
| SU_HO_FOETAL_FEMUR_C5_ENDOTHELIAL_CELL | KIRC | 112 | 4.70E-52 |

Note: Only the top 20 most significant gene sets for each cancer type are displayed.

**Supplementary Table S15.** Independent validation datasets with recurrence information

| Cancer type | Project name | Number of patients | links |
| --- | --- | --- | --- |
| BRCA | METABRIC | 348 | <https://www.cbioportal.org/datasets> |
| COAD | SILU | 1975 |  |
| KIRC | CPTCA | 221 |  |

**Supplementary Table S16.** Genes associated with cancer recurrence identified by Cox regression

| Gene symbol | ENSEMBL ID | Cancer type |
| --- | --- | --- |
| GLUL | ENSG00000135821 | BRCA |
| CCL19 | ENSG00000172724 | BRCA |
| MORC2 | ENSG00000133422 | COAD |
| PYCR2 | ENSG00000143811 | COAD |
| LGALS4 | ENSG00000171747 | COAD |
| COX5A | ENSG00000178741 | COAD |
| DDAH2 | ENSG00000213722 | COAD |
| PHGR1 | ENSG00000233041 | COAD |
| KLF6 | ENSG00000067082 | KIRC |
| NME4 | ENSG00000103202 | KIRC |
| PDCD5 | ENSG00000105185 | KIRC |
| UFSP2 | ENSG00000109775 | KIRC |
| FAAH | ENSG00000117480 | KIRC |
| PGF | ENSG00000119630 | KIRC |
| RAMP3 | ENSG00000122679 | KIRC |
| FKBP10 | ENSG00000141756 | KIRC |
| RGS12 | ENSG00000159788 | KIRC |
| PRDX2 | ENSG00000167815 | KIRC |
| UGT2B7 | ENSG00000171234 | KIRC |
| CES3 | ENSG00000172828 | KIRC |
| SAA1 | ENSG00000173432 | KIRC |
| BANF1 | ENSG00000175334 | KIRC |
| TCN2 | ENSG00000185339 | KIRC |
| EIF4EBP1 | ENSG00000187840 | KIRC |
| MT1F | ENSG00000198417 | KIRC |

**Supplementary Table S17.** Differentially expressed genes in three phenotypes

| Phenotype | Cancer type | No. of DEGs | No. recaptured DEGs | No. recaptured DEGs (same direction) |
| --- | --- | --- | --- | --- |
| Cancer/  Normal | BRCA | 877 | 862 | 862 |
|  | COAD | 854 | 836 | 836 |
|  | KIRC | 875 | 863 | 863 |
| Stage | BRCA | 184 | 159 | 158 |
|  | COAD | 265 | 152 | 152 |
|  | KIRC | 657 | 601 | 601 |
| Grade | BRCA | 967 | 967 | 967 |
|  | KIRC | 751 | 706 | 706 |

**Supplementary Table S18.** Differentially expressed genes between Scissor+ spots and all other cancer-cell spots.

| Gene | p_val | avg_log2FC | p_val_adj |
| --- | --- | --- | --- |
| JCHAIN | 3.71E-45 | -4.27 | 3.62E-42 |
| SCUBE2 | 1.47E-136 | -3.93 | 1.43E-133 |
| AGR2 | 1.44E-213 | 3.67 | 1.41E-210 |
| NEDD4L | 7.25E-93 | -2.98 | 7.08E-90 |
| GASK1B | 2.20E-52 | -2.64 | 2.15E-49 |
| SLC44A4 | 2.86E-165 | -2.60 | 2.79E-162 |
| MRPS30 | 2.66E-188 | 2.55 | 2.60E-185 |
| PRR15 | 4.60E-95 | -2.54 | 4.49E-92 |
| ZG16B | 1.96E-187 | -2.45 | 1.91E-184 |
| FBLN1 | 3.19E-190 | 2.41 | 3.12E-187 |
| PCSK6 | 6.26E-116 | -2.40 | 6.12E-113 |
| COL14A1 | 1.87E-222 | 2.37 | 1.82E-219 |
| KIF13B | 7.47E-62 | -2.33 | 7.30E-59 |
| EVL | 1.46E-187 | -2.32 | 1.43E-184 |
| PDCD4 | 4.60E-19 | -2.25 | 4.49E-16 |
| HLA-DPA1 | 2.16E-129 | -2.16 | 2.11E-126 |
| HMGB3 | 1.55E-182 | 2.15 | 1.52E-179 |
| SELENOP | 1.44E-198 | 2.12 | 1.40E-195 |
| ALDH2 | 1.66E-125 | -2.10 | 1.62E-122 |
| HLA-DQB1 | 3.96E-95 | -2.08 | 3.87E-92 |
| FN1 | 5.87E-147 | 2.07 | 5.73E-144 |
| AFF3 | 2.21E-88 | -2.05 | 2.16E-85 |
| QDPR | 1.82E-95 | -2.04 | 1.78E-92 |
| GFRA1 | 2.17E-152 | 2.04 | 2.12E-149 |
| SMIM14 | 4.30E-107 | -2.03 | 4.20E-104 |
| SH3BGRL | 3.71E-170 | -2.03 | 3.62E-167 |
| HLA-DQA1 | 4.47E-79 | -2.02 | 4.37E-76 |
| GJC3 | 1.86E-52 | -2.02 | 1.82E-49 |
| CD74 | 1.40E-151 | -1.96 | 1.37E-148 |
| SEMA3C | 3.73E-150 | 1.96 | 3.64E-147 |
| IGF1R | 8.52E-197 | 1.95 | 8.32E-194 |
| TFF3 | 2.12E-172 | -1.95 | 2.07E-169 |
| RHOB | 2.33E-28 | -1.94 | 2.27E-25 |
| NBL1 | 6.90E-132 | -1.92 | 6.74E-129 |
| TBC1D9 | 2.70E-173 | -1.89 | 2.64E-170 |
| SERPINI1 | 2.30E-55 | -1.88 | 2.25E-52 |
| LURAP1L | 4.43E-126 | 1.88 | 4.33E-123 |
| FBP1 | 1.87E-118 | -1.86 | 1.83E-115 |
| ATP1B3 | 5.79E-141 | 1.86 | 5.66E-138 |
| CAMK2N1 | 5.00E-79 | -1.84 | 4.89E-76 |
| SNRPN | 5.30E-76 | -1.81 | 5.18E-73 |
| TMEM25 | 2.97E-71 | -1.79 | 2.90E-68 |
| MAGED2 | 1.21E-183 | -1.79 | 1.18E-180 |
| HLA-DRA | 1.86E-113 | -1.75 | 1.82E-110 |
| CAPN8 | 9.07E-61 | -1.73 | 8.86E-58 |
| KIF12 | 2.33E-72 | -1.73 | 2.27E-69 |
| ABAT | 1.95E-60 | -1.72 | 1.90E-57 |
| HLA-DPB1 | 1.06E-87 | -1.72 | 1.03E-84 |
| LMO4 | 3.87E-48 | -1.68 | 3.79E-45 |
| CDK1 | 2.51E-113 | 1.67 | 2.46E-110 |

Note: Only the top 50 genes with the highest absolute log2 fold change are displayed.

**Supplementary Table S19.** scRNA-seq datasets as deconvolution references

| Cancer type | Cell types | Cells | Link |
| --- | --- | --- | --- |
|  |  |  |  |
| BRCA | B_cells/Cancer_epithelial/Myeloid/Plasmablasts/Stromal/T_cells | 100065 | <https://singlecell.broadinstitute.org/single_cell/study/SCP1039/a-single-cell-and-spatially-resolved-atlas-of-human-breast-cancers> |
| COAD | B_cells/Epithelial_cells/Mast_cells/Myeloids/Stromal_cells/T_cells | 27414 | <https://www.ncbi.nlm.nih.gov/geo/query/acc.cgi?acc=GSE144735> |
| KIRC | B_cells/Endothelial_cells/Macrophages/Mast_cells/NK_cells/T_cells/Collecting_duct_epithelial_cells/Erythrocytes/Malignant_cells/Myofibroblasts/Pericytes/Tubular_epithelial&malignant_cells | 33537 | <https://www.ncbi.nlm.nih.gov/geo/query/acc.cgi?acc=GSE224630> |
